# Dataset of materia medica in Sowa Rigpa: Tibetan medicine botanicals and Gawé Dorjé’s classification system

**DOI:** 10.1016/j.dib.2020.106498

**Published:** 2020-11-04

**Authors:** Rigdzin Wangyal, Tawni Tidwell, Wüntrang Dhondrup, Tséwang Yungdrung, Gönpo Dhondrup, Qingxiu He, Yi Zhang

**Affiliations:** a School of Tibetan Medicine, Gansu University of Traditional Chinese Medicine, Hezuo 747000, China; b Center for Healthy Minds, University of Wisconsin-Madison, 625W. Washington Ave, Madison, WI 53711, United States; c College of Pharmacy, Qinghai Nationalities University, Xining 810007, China

**Keywords:** Materia medica, Botanical identification, Tibetan medicine, Sowa Rigpa, Ethnobotanical classification, Ethnopharmacology

## Abstract

This article provides the most updated dataset of Latin botanical identifications for the materia medica in Tibetan medicine, known as Bö Luk Sowa Rigpa (Tib. *Bod lugs gso ba rig pa*), or the “Tibetan knowledge field of healing,” often denoted in English simply as Sowa Rigpa. As one of the major scholarly Asian traditional medical systems, Sowa Rigpa is the principal health resource for populations across Tibetan regions of China, Mongolia, Bhutan, Nepal, India, and culturally-related areas of Russia. The geography represented by this medicinal plant dataset extends across the entire Tibetan plateau, its adjacent ranges, the wider transregional Himalayas, central Asia and much of the Indian subcontinent. Data collection drew from textual analysis of the seminal works of the Tibetan medical canon, including the *Four Medical Treatises, Crystal Orb and Rosary* among others; as well as the contemporary definitive work *Stainless Crystal Mirror of Materia Medica* by Gawé Dorjé. Study authors applied the same classification system as Gawé Dorjé, yet reanalyzed specimens according to a database cataloging research on regional herbarium botanical specimens, geographic distributions and regional plant chemistry studies, and confirming proper identification with the most current modern botanical taxonomies. Subsequently, almost 700 of the most commonly used materia medica were selected for compilation. Thus, this dataset represents updated botanical identifications and confirmations from both early and contemporary sources. Botanical specimen names were entered into spreadsheet format with Gawé Dorjé’s categories listed alongside Deumar Tendzin Püntsok's early standard. Enclosed raw data are written in Unicode Tibetan font to retain fidelity to entries in the classical texts, with parallel columns in standard Wylie Tibetan transliteration and phonetic transcription. Latin botanical names are updated for each materia medica specimen using Kew's Medicinal Plant Names Services (Kew-MPNS) with missing entries supplied by World Flora Online (WFO) and Flora of China (FoC). This dataset is the first publicly available comprehensive ethnobotanical identification of Sowa Rigpa materia medica with Latin binomial nomenclature. This dataset was developed to inform botanical and pharmacological analysis of the Tibetan medical materia medica repertoire as well as make comparative analyses of related materia medica in other Asian medical systems.

Specifications Table

**Table:** 

Subject	Complementary and Alternative Medicine
Specific subject area	Materia medica, medical botany, Tibetan medicine, Sowa Rigpa, botanical identification, classification
Type of data	Table
How data were acquired	Data obtained from Tibetan medical classical root texts and commentaries, namely, Yutok Yönten Gönpo's *Four Medical Treatises* (*Rgyud bzhi*) [1], Deumar Tendzin Püntsok's *Stainless Crystal Rosary* (*Shel gong shel phreng*) [2], and Gawé Dorjé’s *Stainless Crystal Mirror of Materia Medica* (*Sman gyi ’khrung dpe dri med shel gyi me long*) [3]; as well as contemporary botanical identification studies, informatics analytics of botanical data and compound analysis, and open access databases of accepted taxa of plant families. Data entered into Excel spreadsheet.
Data format	Raw, analyzed and categorized.
Parameters for data collection	Data are classified and organized by Latin botanical and ethnobotanical category and written in Unicode Tibetan font, Wylie Standardized Tibetan transliteration, phonetic transcription and identified Latin botanical binomial name.
Description of data collection	Data obtained from textual analysis of various Tibetan medical classics and contemporary compendiums, including *Four Medical Treatises, Stainless Crystal Rosary*, and *Stainless Crystal Mirror of Materia Medica*; then almost 700 of the most commonly used materia medica were selected for compilation; reanalyzed according to databases cataloging research on regional herbarium botanical specimens, geographic distributions and regional plant chemistry studies; updated according to modern botanical taxonomy to confirm accepted name; and re-categorized by ethnobotanical class.
Data source location	Ethnic Medicine Academic Heritage Innovation Research Center,
	Chengdu University of Traditional Chinese Medicine,
	Chengdu 611137 PR China
Data accessibility	With the article

## Value of the Data

This dataset provides researchers in the traditional medical and ethnopharmacological fields access to botanical materia medica identifications most important to Sowa Rigpa formulas, based on the original Tibetan medical source data widely considered authoritative in the Sowa Rigpa tradition.

This data provides important contributions to researchers working with any major scholarly Asian medical tradition as well as other traditional botanical medical systems more broadly. Assessing similarities and distinctions across materia medica of traditions such as Indian and Thai Ayurveda, traditional Chinese medicine, Unani and various other Greco-Arabic traditions, provides important insights into therapeutic repertoires for specific disease classes, medicine compounding principles and chemical and pharmacological transformations used. It also provides insights into a shared history of trade through contributions of the Silk Route and other forms of shared material knowledge and exchange.

This data is also significant for research investigating the scope of Tibetan medical pharmacology, its categories, its most potent compounds, the degree of endemic species used, and chemical indications of what conditions might benefit most significantly from such a medical botanical repertoire.

Providing researchers with the standard classification system from the traditional authority of materia medica in Lhasa, this data informs detailed comparisons to regional variations and local substitutes that have developed across the Tibetan plateau as well as extending into the wider trans-Asian regions in which Sowa Rigpa still provides major healthcare, medical and ethnopharmacological contributions.

This data facilitates research analyzing distinctions across materia medica, within compounded formula relationships and between related classification hierarchies in Tibetan pharmacology that implicate differential compound recognition. It also supports investigations targeting potential therapeutic benefits from botanical compounds; as well as analysis of compounding theory according to the specific botanical ingredients identified.

This data facilitates research assessing frequencies, abundance patterns and ecozones relevant to these materia medica and analyses of ecological niches, soil characteristics, and micro-climates in which such materia medica develop. By linking geospatial distributions of these species, this data allows researchers to analyze the degree to which climate change, land use patterns and habitat destruction may impact the sustainability of these medicinal resources and the viability of the botanical tradition of this medical system more broadly. Such analyses help medical and pharmacological researchers to identify potential future substitutes through compound analysis.

## 1 Data Description

This dataset (Table 1 Supplemental materials) provides the Latin botanical identifications for the materia medica in the Tibetan medical tradition, also known as Sowa Rigpa (*Gso ba rig pa*) [4], which is one of Asia's great scholarly traditional medical systems alongside Chinese medicine (TCM), Ayurveda and Unani [5]. Emergent between the seventh and twelfth centuries CE, it spread most significantly in the thirteenth and seventeenth centuries CE, transmitting across the Tibetan plateau, Mongolia and the Himalayas. Today, it still provides a central healthcare resource for populations across a large region of Asia, including Tibetan regions of China, Mongolia, Bhutan, Nepal, India, and culturally-related areas of Russia. Though influenced significantly by indigenously Tibetan foundational contributions, it integrates medical knowledge from Indian, Chinese, Persian, and Central Asian sources. The pharmacology of Sowa Rigpa comprises complex multi-compound, rigorously processed formulas of a wide range of herbs, minerals, metals and animal product ingredients [6]. Formulas can range from several ingredients to upward of 140 ingredients in a single pill.

This dataset provides the most rigorously analyzed identifications and classifications of Sowa Rigpa materia medica botanicals used in Tibet to date, following Gawé Dorjé’s [3] classification system (e.g., [7]). The data update his most recent identifications [3]; re-analyze them according to databases cataloging research on regional herbarium botanical specimens, geographic distributions and regional plant chemistry studies; confirm the most current accepted names; and re-categorize them by ethnobotanical class. This dataset results from an investigation of the proper identifications for 700 of the most commonly used specimens in the Tibetan materia medica. This botanical knowledge provides the foundational medicinal plant resource for Tibetan medicine as applied across the three regions of Tibet—Central Tibet and the two eastern regions of Kham and Amdo that stretch across present-day Qinghai, Gansu, Sichuan and Yunnan provinces, as well as the Tibetan Autonomous Region (TAR). It also provides a cross-reference to other compilations in Tibet and those focused on Sowa Rigpa botanicals used in India, Nepal, Bhutan and Mongolia (e.g., [8]), facilitating rich comparisons of the tradition's botanical plasticity [9]. The geography represented by this medical flora encompasses a land mass spanning about 2.5 million km^2^, equivalent to the size of central Europe. Historic trade along the Silk Route provided a major resource for some of the most potent materia medica principally used in Sowa Rigpa and facilitated access to these important medicinal materials across the various regions of its scope on the Tibetan plateau, north India, Nepal, Bhutan, Mongolia, and areas of Russia, such as Buryatia, Kalmykia and Tuva. Likewise, representations from these various regions also provide significant contributions to the materia medica herein due to these rich systems of exchange. However, the principal regions from which the greatest specimen numbers of Sowa Rigpa materia medica derive are the Tibetan plateau itself, followed by the Indian subcontinent, subtropical areas of mainland China, and eastern parts of central Asia.

The Tibetan plateau and the wider transregional Himalayas alone comprise a diverse range of ecosystems from tropical zones at the bases of mountain subranges to permanent ice, snow and stark, glaciated terrain at the highest elevations. These wide-ranging climates as well as varied rainfall, altitude, geologies and soils, also support an extensive and diverse range of flora and fauna, including highly distinct forms of plant and animal communities. The Himalayan region contains almost 10,000 plant species comprising 2.5% of the world's angiosperm diversity from which 4000 species are endemics [10]. Ongoing research is interested in how Himalayan flora achieved such high endemic plant diversity from the initial immigrant taxa that populated the region, with initial hypotheses investigating the role of major periodic geophysical upheavals that disrupted ecosystem stability and injected biological diversification [10].

Ethnobotany and its sister field ethnoecology look at how a population perceives, manages and uses its botanical and ecological resources. As the primary field of naturalist knowledge for this cultural region, Sowa Rigpa understandings of materia medica can provide key insights into the characteristics, ecological relationships and sustainable management of these environmental and health resources. Craig and Glover [11] describe medicinal plants as ‘biocultural objects’ situated within social and cultural fields of activity and knowledge that signal their fundamental utility. They build on Pordié’s notion [13] that plants are themselves expressions of culture in that they convey the varying values attached to them by the different human communities and actors with which they come into contact, conflict or systems of exchange. In this context, these plants are based in a particular kind of knowledge system related to etiology, diagnosis and therapeutic consumption in which they become the means through which treatment is enacted, illness is addressed, and various psychophysiological activities are mobilized.

These medicinal botanicals also provide rich sources of pharmacological knowledge as demonstrated by growing research on their functional activities and properties (e.g., [12], [14], [15]), as well as the growing clinical research [16], [17]. Previous work on Sowa Rigpa geomedicinals [18] provides insight into the diverse scope of Sowa Rigpa materia medica engagement with the natural world.

Although this dataset does not elaborate on the classification systems used for the materia medica in Sowa Rigpa, it notes the major traditional classes for each specimen alongside the modern botanical family. Thus, this data help facilitate inquiry into ethnoclassification, which is of particular interest to the fields of anthropology and ethnobotany/ethnoecology [[7], [19], [20], [21]], and ethnopharmacology [[6],[12], [14], [15]]. It also helps distinguish applied practices of substitution, local variations and formulation variation based on botanical plasticity and local healthcare needs used in Sowa Rigpa of the materia medica that are primarily regional and contextual [[7], [22], [24]].

## 2 Experimental Design, Materials, and Methods

This dataset develops work that is the product of many years of effort, experience, and analysis, as well as consultation with experts in numerous fields of traditional Tibetan medicine, botany, ethnobotany and pharmacological analysis. Most significantly, it draws upon the work of Gawé Dorjé, medical and pharmacology professor at University of Tibetan Medicine, who has painstakingly compiled and published [3] medicinal substances across the traditional medical texts in Tibet and requested the current authors to conduct a full reanalysis according to modern botanical taxonomies and confirm the proper identification of 700 of the most commonly used materia medica specimens in Sowa Rigpa.

The textual source for the reanalysis is *Stainless Crystal Mirror of Materia Medica* [3], which has become the single most important reference text for traditional identification and analysis of medicinal plants in Tibetan medical hospitals and clinics, schools, medical houses and monastic medical colleges across the Tibetan plateau. Revered by great scholars of Tibet, such as Troru Tsenam, Jampa Trinlé and Aku Tenko, it is recognized as the highest commentarial authority on the topic. Gawé Dorjé’s texts [3] result from detailed review of both the classical and contemporary texts on materia medica in the Tibetan medical corpus. As such, beyond the Tibetan medical classic *Four Medical Treatises* [1] as the foundational canon of Sowa Rigpa, the primary source text or definitive foundational resource for writing his illustrated reference text is Deumar Tendzin Püntsok's *Stainless Crystal Rosary* (*Dri med shel phreng*) [2], which is the most detailed classic text explicating traditional materia medica in Sowa Rigpa. A few of the more significant texts that informed Gawé Dorjé’s exhaustive review, but not as clearly elucidated as *Stainless Crystal Rosary* include: *Venerable Tara's Materia Medica* (*Rje btsun sgrol ma'i sngo ’bum*) in *A Treatment Compendium of Precious Materia Medica* (*Gso dpyad rin po che ’khrungs dpe*), *Manjugosha Materia Medica* (*’Jam dbyangs sngo ’bum*) (2005), *Yutok's Materia Medica* (*G.yu thog sngo ’bum*), *Illuminating Ambrosial Lamp of Medicinal Herbs* (*Sman ngo gsal byed bdud rtsi'i sgron me*), *Sun of Etymological Meaning* (*Tshig don nyi ma*); and *Blue Beryl* (*BaiDUr+ya sngon po*).

Through the review, Gawé Dorjé confirmed his sources as consistent with these other definitive works. His-texts provide a comprehensive compilation of materia medica data from these transmitted texts, practices and oral instructions, while simultaneously developing ecological descriptions of each specimen making the information highly accessible and relevant to research interlocutors. He has sought out and confirmed traditional identifications of physical specimens of the materia medica with preeminent scholars and practitioners of medicine compounding throughout Tibet, and as such has developed the definitive work for materia medica descriptions, growth patterns, ecological niches and identifications in the traditional classical system. Nevertheless, developing such a definitive work has some limitations. For one, the work assigns standardized identifications of the accepted Latin botanical names for these specimens according to a more centralized geographic region. Compared to the larger scope of Sowa Rigpa influence, this approach can collapse the heterogeneity with which the traditional Sowa Rigpa identification permits a given materia medica specimen to have acceptable regional identification variations and distinctions in potency relevant to locality, evading political economies of regulation. Yet, Gawé Dorjé’s work attempts to integrate regional variations and modifications within the text and allows for previous descriptions of superior (*mchog*) and inferior (*dman*) types to simply be enumerations within the larger category of the traditionally described plant specimen. Previous distinctions between schools of thought in plant recognition such as the Jang and Zur schools contribute variations of perspective within the text instead of conflicting paradigms to which one must align.

In Gawé Dorjé’s classification [3], he systematized materia medica into eight major divisions that follow some of the classical classification modes, but also integrate several from contemporary taxonomical approaches. His-eight divisions are as follows: (1) precious substances (*rin po che'i sman sde*); (2) non-precious geologically-derived substances (*sa rdo'i sman sde*); (3) salt classes (*tshwa sna'i sman sde*); (4) extracts (*rtsi sman gyi sde*); (5) woody plants (*shing sman gyi sde*); (6) shrubs and herbaceous plants (*sngo ldum gyi sman sde*); (7) domesticated grains, legumes and pulses (*’bru yi sman sde*); and (8) fauna-derived substances (*srog chags kyi sman sde*). Within the category of precious medicines, he retained the classic subcategories of meltable (*bzhu ba'i khams*) and un-meltable substances (*mi bzhu ba'i khams*). The fauna further delineate into subcategories of mammals (*’o ’thung srog chags*; literally, “animals which drink milk”); birds (*bya rigs*); and insects, reptiles, amphibians and fish (*’bu srin gyi sde*).

Gawé Dorjé identified 95 compounds in the precious substances category; 120 substances as non-precious geologically-derived substances; 27 types of salts; 23 types of extracts; 173 specimens in the woody plants category; 488 specimens in the shrubs and herbaceous plants category; 36 medicinal substances classified as domesticated grains, legumes and pulses; and 139 species as fauna from which medicinal substances are derived for 468 types of medicines recognized from those species. In total, Gawé Dorjé enumerates over 1430 types of medicinal substances in his text [3].

For years Gawé Dorjé has been interested in differentiating specimens according to modern botanical taxonomic identification so as to elucidate the nuance in the Tibetan medical classification system that had previously incurred confusion in conversations with Western researchers when various distinctly identified specimens in the Tibetan taxonomical system fell under a single species or Latin nomenclature in the Western taxonomical system (see, for instance, a single *Terminalia chebula* Retz botanical identification for multiple types of *arura* (*a ru ra*) from the Sowa Rigpa system). Dorjé was also interested in establishing clarifications on debates within the Tibetan medical field of specific materia medica identifications and elucidating distinctions for colleagues recognizing similar species and sub-species in traditional Chinese medicine and Ayurveda. His-text even includes the accepted Chinese name for each specimen since his main non-Tibetan interlocutors are Chinese medical physicians and researchers. This has the potential to create equivalences with TCM materia medica which may not be identical in terms of sourcing, processing, traditional classification and description. However, the attempt, again, is at creating bridges of discourse in both clinical and research settings. The dataset provided herein does not include nor reanalyze the Chinese identifications since the aim of this dataset is to provide the accepted Latin botanical names for the Tibetan identifications to an English-speaking research and clinical community. Due to his focus on providing a resource that facilitated rigorous engagement with the research community as well as a comprehensive materia medica compilation for practioners, Gawé Dorjé analyzed the materia medica literature thoroughly and substantiated his definitive identifications with related source texts and scholars, as well as his own analysis and consultation with relevant botanical, biological and geological experts.

It is in the endeavor of consultation with the current authors that Gawé Dorjé requested an official dataset of identifications be developed in accordance with the most recent ethnobotanical and ethnopharmacological research as well as the currently accepted names of Latin binomial nomenclature. With the founding of a new research department at the Ethnic Medicine College of Chengdu University of Traditional Medicine, the current authors were able to draw upon a comprehensive database called the Northwest Minority Medical Database (*Byang nub grangs nyung mi rigs gso rig gzhi grangs mdzod*), which stores medicinal compound and pharmacological data from the various research projects conducted by faculty, students and researchers of the department as well as external researchers. With Gawé Dorjé’s rigorous methodology in traditional identification and confirmation of the materia medica with scholars across Tibet and initial Latin botanical identifications, the research team was able to reanalyze the data using keyword phrases across 410 ancient Tibetan medical texts, over 1500 compounded formulas and more than 3000 medicinal compounds to confirm the proper Latin botanical identifications. The research team also drew upon a database that catalogues proper identifications through regional herbarium botanical specimens (as recommended for identification and authentication studies [23]), geographic distributions and regional plant chemistry studies; and confirmed proper identification with the most current available modern botanical taxonomies. Such methodology has provided extensive new investigation possibilities in the field of Tibetan medical informatics [24].

The coauthors identified 690 materia medica specimens that represent the most commonly used specimens in Tibetan medicinal formulas. This assessment was done through frequency analysis of ingredients of formulas primarily used in Qinghai, Gansu, Sichuan, Yunnan, and the hospitals and clinics of Lhasa and other centers in the Tibetan Autonomous Region. These specimens were confirmed with Latin botanical identifications to species level. The updated Latin botanical names for the dataset were provided for each materia medica specimen using Kew's Medicinal Plant Names Services (Kew-MPNS) [25] with missing entries supplied by Kew's World Checklist of Selected Plant Families (WCSP) [26], World Flora Online (WFO) [27], and Flora of China (FoC) [28].

Kew-MPNS is an online database managed by Kew Royal Botanical Gardens of medicinal plant data and medical citations of literature published under available pharmaceutical, drug, common and scientific plant names that are continuously validated and updated through embedded persistent digital identifiers that also correct for spelling, reconcile detected ambiguities and duplications. Kew-MPNS provides a global database of modern plant taxonomy for medicinal plants. WCSP is a live and daily updated compiled database of peer reviewed data on all accepted taxa of plant families by the Kew Royal Botanic Gardens. Newly published names are automatically added from the International Plant Names Index (IPNI) [29] but only visible on the website after a one-year editing phase. Kew-MPNS draws from unpublished data of the WCSP, and thus provides a more updated source compared to data on the publicly available version of the WCSP.

WFO is also an open-access, online database of the world's plant species built out of the updated Global Strategy for Plant Conservation (GSPC) of the United Nations Convention on Biological Diversity in 2010. It includes existing knowledge and published floras, checklists and revisions. The Plant List (TPL) [30] was built out of the 2002–2010 GSPC to provide a working list of all known plant species produced by the botanical community. It was developed to provide the currently accepted name used in preference to refer to the species, subspecies, variety or form; the author or authors credited with publishing that name; the place and date of the original publication in which the name was supplied; a reference to the source database supplying the name record that recorded the opinion that it is an accepted name with, where possible, a link to that record in the source database; and other names (synonyms) considered to refer to that species. TPL provided the taxonomic backbone for the WFO but has been static since 2013. WFO now provides the most updated source.

The FoC database [26] is a collaborative database of vascular plants of China maintained by Beijing's Science Press and Missouri Botanical Garden. Additional Chinese editorial collaborators include CAS Institute of Botany (Beijing), the Kunming Institute of Botany, the Jiangsu Institute of Botany (Nanjing), and the South China Botanical Garden (Guangzhou); and non-Chinese editorial collaborators including Harvard University Herbaria, the California Academy of Sciences, the Smithsonian Institution, the Royal Botanic Garden Edinburgh, Kew Royal Botanic Gardens, and the Muséum National d'Histoire Naturelle (Paris). Though it is a specialized database compiling published data on plants of China, its entries are not as frequently updated as those of the MPNS, WCSP and WFO.

Thus, the author team conducted the process of confirming the accepted botanical names in order of the databases with the most updated entries: Kew-MPNS, followed by WCSP, WFO, and FoC. Most names in the dataset were confirmed by MPNS and WFO. Assessing the chronological bibliography and publication of names relied on references from the Plants of the World Online (POWO) [31] and International Plant Names Index (IPNI) databases, respectively. POWO and IPNI are bibliographic resources which provide full original publication details for a recognized botanical name.

MPNS and WFO draw from IPNI for the published recognized botanical name, while additionally incorporating current opinions from experts on the relevant taxonomic groups. Taxonomic revisions of entire groups that occur because of previous errors in published names, misidentified specimens and propagation of old names. Thus, this dataset represents the dynamic nature of botanical taxonomy that includes constant developments and updates from newfound sequencing data. However, since botanical taxonomy still prioritizes the oldest accurately published name or specimen, this list also represents the conservatism of the field.

Table 1 (in Supplemental materials) represents the specimens originating in the Sowa Rigpa canon. The accompanying raw data (in Supplemental materials) provides the comprehensive list of the materia medica with names in classical Tibetan script, their Latin identifications, standardized transliteration of the Tibetan, and standardized transcription of phonetic pronunciation.

## CRediT Author Statement

**Rigdzin Wangyal**: collected, collated, analyzed and interpreted the data. **Tawni Tidwell**: collected, analyzed and interpreted the data and wrote and revised the manuscript. **Rigdzin Wangyal and Tawni Tidwell**: updated and confirmed the Latin botanical names using the described databases. **Rigdzin Wangyal, Wüntrang Dhondrup, Tséwang Yungdrung, Gönpo Dhondrup, Qing Xiu He, and Yi Zhang**: conceived and designed the study, and revised the manuscript. **Yi Zhang**: supervised the research group. The final version of the manuscript was read and approved by all authors.

## Declaration of Competing Interest

The authors declare that they have no known competing financial interests or personal relationships which have, or could be perceived to have, influenced the work reported in this article.

## Appendix Supplementary materials

Image, application 1

Image, application 2

## Footnotes

Supplementary material (Table 1 and raw data) associated with this article can be found in the online version at doi:10.1016/j.dib.2020.106498
