# Supplementary material for: Dataset of materia medica in Sowa Rigpa: Tibetan medicine botanicals and Gawé Dorjé’s classification system
Source: Data Brief. 2020 Nov 4;33:106498. doi: 10.1016/j.dib.2020.106498 (PMC7689029; doi:10.1016/j.dib.2020.106498)
Supplement: Supplementary file 2 [file mmc2.docx]

Table 1 Botanical materia medica described in the Sowa Rigpa pharmacopoeias

| **Latin Botanical Name (Binomial Nomenclature)**  ***Accepted name at publication of Gawé Dorjé (2018)** | **Latin Botanical Name (Binomial Nomenclature)**  ***Currently accepted name according to MPNS/WFO** | **Botanical family** | **Sowa Rigpa name** (Wylie Standardized Transliteration of Tibetan) | **Sowa Rigpa family**  **(Gawé Dorjé System)** | **Sowa Rigpa family**  **(Deumar Tenzin Puntsok System)** |
| --- | --- | --- | --- | --- | --- |
| *Abelmoschus moschatus* (L.) Medicus. | *Abelmoschus moschatus*Medik. | Malvaceae | so ma ra dza | sngo ldum | sngo sman |
| *Abrus precatorius* Linn. | *Abrus precatorius*L. | Fabaceae | mda' rgyus | shing sman | shing sman |
| *Acanthocalyx* alba (Hand.-Mazz.) M. Connon | *Acanthocalyx alba*(Hand.-Mazz.) M.J.Cannon | Dipsacaceae | byi tsher dkar po | sngo ldum | sngo sman |
| *Aconitum gymnandrum* Maxim. | *Aconitum gymnandrum*Maxim. | Ranunculaceae | 'dzin pa zla bral | sngo ldum | sngo sman |
| *Aconitum kongboense* Lauener | *Aconitum kongboense*Lauener | Ranunculaceae | ra dug dkar po | sngo ldum | sngo sman |
| *Aconitum leiwuqiense* W.T.Wang | *Aconitum leiwuqiense*W.T.Wang | Ranunculaceae | ri bo che'i bong nag | sngo ldum | sngo sman |
| *Aconitum ludlowii* Exell | *Aconitum ludlowii*Exell | Ranunculaceae | bong nga nag po | sngo ldum | sngo sman |
| *Aconitum naviculare*（Brühl）Stapf | *Aconitum naviculare*(Brühl) Stapf | Ranunculaceae | bong nga dkar po | sngo ldum | sngo sman |
| *Aconitum pendulum* Busch | *Aconitum pendulum*N.Busch | Ranunculaceae | brsan dug ser po | sngo ldum | sngo sman |
| *Aconitum polyanthum* (Finet et Gagnep.) Hand.-Mazz. var. *puberulum* W. T. Wang. | *Aconitum polyanthum* (Finet & Gagnep.) Hand.-Mazz. | Ranunculaceae | ra dug pa | sngo ldum | sngo sman |
| *Acorus calamus* L. | *Acorus calamus*L. | Araceae | shu dag | sngo ldum | ldum bu thang sman |
| *Acorus gramineus* Soland. | *Acorus gramineus*Aiton | Araceae | shu dag dkar po | sngo ldum | ldum bu thang sman |
| *Adenophora stenanthina*（Ledeb.）Kitagawa. | *Adenophora stenanthina*(Ledeb.) Kitag. | Campanulaceae | klu bdud g.yu dril ma | sngo ldum | sngo sman |
| *Adhatoda vasica* Nees | *Adhatoda vasica* Nees | Acanthaceae | ba sha ka | sngo ldum | ldum bu thang sman |
| *Adonis coerulea* Maxim. | *Adonis coerulea*Maxim. | Ranunculaceae | rgya rtsi g.yung ba | sngo ldum | ldum bu thang sman |
| *Aegle marmelos* (L.) Correa | *Aegle marmelos*(L.) Corrêa | Rutaceae | bil ba | shing sman | shing sman |
| *Aesculus chinensis* Bunge | *Aesculus chinensis*Bunge | Hippocastanaceae | so cha | shing sman | shing sman |
| *Agaricus benesii*（Pilát）Pilát | *Agaricus benesii*（Pilát）Pilát | Agaricaceae | sha mong smug po | sngo ldum | sngo sman |
| *Agaricus bisporus* (Lange) Sing. | *Agaricus bisporus* (Lange) Sing. | Agaricaceae | dngul sha | sngo ldum | sngo sman |
| *Ajania purpurea* Shih | *Ajania purpurea* C.Shih | Asteraceae | 'khan pa a krong | sngo ldum | ldum bu thang sman |
| *Ajania purpurea* Shih | *Ajania purpurea*C.Shih | Asteraceae | 'khan a krong | sngo ldum | ldum bu thang sman |
| *Ajania tenuifolia* (Jacq.) Tzvel. | *Ajania tenuifolia* Tzvelev | Asteraceae | 'khan dkar | sngo ldum | ldum bu thang sman |
| *Ajuga lupulina* Maxim. | *Ajuga lupulina*Maxim. | Lamiaceae | zin tig | sngo ldum | sngo sman |
| *Ajuga ovalifolia* Bur. et Franch. var. *calantha*（Diels ex Limpricht）C. Y. Wu et C.Chen | *Ajuga ovalifolia* var. *calantha*(Diels) C.Y.Wu & C.Chen | Lamiaceae | klung skyes rta lpags | sngo ldum | sngo sman |
| *Alcea rosea* Linnaeus. | *Alcea rosea*L. | Malvaceae | pho lcam | sngo ldum | ldum bu thang sman |
| *Allium atrosanguineum* Schrenk | *Allium atrosanguineum*Schrenk | Liliaceae | ri sgog | sngo ldum | sngo sman |
| *Allium carolinianum* DC. | *Allium carolinianum*DC. | Liliaceae | ri skyes sgog | sngo ldum | sngo sman |
| *Allium changduense* J. M. Xu | *Allium changduense*J.M.Xu | Liliaceae | brag sgog | sngo ldum | sngo sman |
| *Allium fasciculatum* Rendle | *Allium fasciculatum*Rendle | Liliaceae | klung sgog ke dzi | sngo ldum | sngo sman |
| *Allium fistulosum* L. | *Allium fistulosum*L. | Liliaceae | tsong sgog | sngo ldum | sngo sman |
| *Allium kingdonii* Stearn | *Allium kingdonii*Stearn | Liliaceae | rgya ba sgog pa | sngo ldum | sngo sman |
| *Allium prattii* C. H. Wright ex Hemsl. | *Allium prattii* C.H.Wright | Liliaceae | rug sgog | sngo ldum | sngo sman |
| *Allium przewalskianum* Regel | *Allium przewalskianum*Regel | Liliaceae | 'dzin nag | sngo ldum | sngo sman |
| *Allium sativum* L. | *Allium sativum*L. | Liliaceae | sgog skya | sngo ldum | sngo sman |
| *Allium sikkimense* Baker | *Allium sikkimense*Baker | Liliaceae | bye'u sgog | sngo ldum | sngo sman |
| *Alpinia officinarum* Hance | *Alpinia officinarum*Hance | Zingiberaceae | sman sga | sngo ldum | ldum bu thang sman |
| *Alpinia pumila* Hook. f. | *Alpinia pumila*Hook.f. | Zingiberaceae | sga rgod | sngo ldum | ldum bu thang sman |
| *Amaranthus ascendens* Loisel. | *Amaranthus ascendens* Loisel. | Araliaceae | rgya sne rgod pa | sngo ldum | sngo ldum |
| *Amaranthus caudatus* L. | *Amaranthus caudatus*L. | Araliaceae | rgya sne | sngo ldum | sngo ldum |
| *Amomum compactum* Soland ex Maton | *Amomum compactum*Sol. ex Maton | Zingiberaceae | sug smel | rtsi sman | rtsi sman |
| *Amomum subulatum* Roxb. | *Amomum subulatum*Roxb. | Zingiberaceae | ko la dkar po | rtsi sman | rtsi sman |
| *Amomum tsaoko* Crevost et Lem. | *Amomum tsao-ko*Crevost & Lemarié | Zingiberaceae | ka ko la | rtsi sman | rtsi sman |
| *Amygdalus persica* L. | *Amygdalus persica* L. | Rubiaceae | kham rag sha | shing sman | shing sman |
| *Anaphalis hancockii* Maxim. | *Anaphalis hancockii*Maxim. | Asteraceae | spra g.yung lug bal ma | sngo ldum | sngo sman |
| *Anaphalis nepalensis*（Spreng.）Hand.-Mazz. | *Anaphalis nepalensis* (Spreng.) Hand.-Mazz. | Asteraceae | spra g.yung chung ba | sngo ldum | sngo sman |
| *Anaphalis tibetica* Kitam. | *Anaphalis tibetica*Kitam. | Asteraceae | spr rgod chung ba | sngo ldum | sngo sman |
| *Anaphalis xylorhiza* Sch.-Bip. ex Hook. f. | *Anaphalis xylorhiza*Sch.Bip. ex Hook.f. | Asteraceae | spra rgod rgyab skya | sngo ldum | sngo sman |
| *Androsace mariae* Kanitz | *Androsace mariae* Kanitz | Primulaceae | sga tig nag po | sngo ldum | sngo sman |
| *Androsace stenophylla* (Petitm.) Hand.-Mazz. | *Androsace stenophylla*(Petitm.) Hand.-Mazz. | Primulaceae | sga tig smug po | sngo ldum | sngo sman |
| *Androsace strigillosa* Franch | *Androsace strigillosa*Franch. | Primulaceae | dwags po sga tig | sngo ldum | sngo sman |
| *Androsace tapete* Maxim. | *Androsace tapete* Maxim. | Primulaceae | spang a krong | sngo ldum | sngo sman |
| *Androsace wardii* W. W. Smith | *Androsace wardii*W.W.Sm. | Primulaceae | sga tig khra bo | sngo ldum | sngo sman |
| *Anemone demissa* Hook. f. et Thoms | *Anemone demissa*Hook.f. & Thomson | Ranunculaceae | srub sngon | sngo ldum | sngo sman |
| *Anemone rivularis*Buch.-Ham. ex DC. | *Anemone rivularis*Buch.-Ham. ex DC. | Ranunculaceae | srub ka | sngo ldum | sngo sman |
| *Anisodus luridus* Link et Otto | *Anisodus luridus*Link | Solanaceae | khra gshog pa | sngo ldum | ldum bu thang sman |
| *Anisodus tanguticus* （Maxim.）Pascher | *Anisodus tanguticus*(Maxim.) Pascher | Solanaceae | thang phrom nag po | sngo ldum | ldum bu thang sman |
| *Anthriscus nemorosa*（M．Bieb.）Spreng. | *Anthriscus nemorosa*(M.Bieb.) Spreng. | Apiaceae | ba lang lca ba | sngo ldum | sngo sman |
| *Taphrospermum altaicum* C.A.Mey. | *aphrospermum altaicum* C.A.Mey. | Brassicaceae | sha sha rub rub | sngo ldum | sngo sman |
| *Aquilaria agallocha* Roxb. | *Aquilaria agallocha* Roxb. | Thymelaeaceae | ar nag | shing sman | shing sman |
| *Aquilaria sinensis* (Lour.) Spreng. | Aquilaria sinensis (Lour.) Spreng. | Thymelaeaceae | ar skya | shing sman | shing sman |
| *Arctium lappa* L. | *Arctium lappa*L. | Asteraceae | byi bzung | sngo ldum | ldum bu thang sman |
| *Areca catechu* L. | *Areca catechu*L. | Arecaceae | go yu | shing sman | shing sman |
| *Arenaria kansuensis* Maxim. | *Arenaria kansuensis*Maxim. | Caryophyllaceae | a krong dkar po | sngo ldum | ldum bu thang sman |
| *Arisaema flavum* (Forsk.) Schott | *Arisaema flavum* (Forssk.) Schott | Araceae | dwa ba | sngo ldum | sngo sman |
| *Aristolochia griffithii* HooK. f. et Thoms. ex Duchartre. | *Aristolochia griffithii*Hook.f. & Thomson ex Duch. | Aristolochiaceae | ba le ka | sngo ldum | sngo sman |
| *Aristolochia moupinensis* Franch. | *Aristolochia moupinensis*Franch. | Aristolochiaceae | rgya ba le | shing sman | shing sman |
| *Armillaria luteo-virens*（Aalb.et Schw:Fr.）Sacc. | *Armillaria luteo-virens*（Aalb.et Schw:Fr.）Sacc. | Tricholomataceae | gser sha | sngo ldum | sngo sman |
| *Artemisia annua* Linn. | *Artemisia annua*L. | Asteraceae | 'khan nag | sngo ldum | ldum bu thang sman |
| *Artemisia conaensis* Ling et Y. R. Ling. | *Artemisia conaensis*Ling & Y.R.Ling | Asteraceae | tshar bong smug po | sngo ldum | ldum bu thang sman |
| *Artemisia desertorum* Spreng. | *Artemisia desertorum*Spreng. | Asteraceae | tshar bong | sngo ldum | ldum bu thang sman |
| *Artemisia dubia* Wall. ex Bess | *Artemisia dubia*L. ex B.D.Jacks. | Asteraceae | phur nag | sngo ldum | ldum bu thang sman |
| *Artemisia hedinii* Ostenf. et Pauls. | *Artemisia hedinii*Ostenf. | Asteraceae | zangs rtsi nag po | sngo ldum | sngo sman |
| *Artemisia moorcroftiana* Wall.ex DC. | *Artemisia moorcroftiana*Wall. | Asteraceae | 'khan dmar | sngo ldum | ldum bu thang sman |
| *Artemisia scoparia* Waldst. et Kit. | *Artemisia scoparia*Waldst. & Kitam. | Asteraceae | tshar bong nag po | sngo ldum | ldum bu thang sman |
| *Artemisia sieversiana* Ehrhart ex Willd. | *Artemisia sieversiana*Ehrh. | Asteraceae | 'khan pa | sngo ldum | ldum bu thang sman |
| *Artemisia vestita* Wall.ex Bess | *Artemisia vestita*Wall. ex Besser | Asteraceae | phur nag lo sib | sngo ldum | ldum bu thang sman |
| *Artemisia younghusbandii* J.R.Drumm.ex Pamp. | *Artemisia younghusbandii*J.R.Drumm. ex Pamp. | Asteraceae | phur dkar | sngo ldum | ldum bu thang sman |
| *Asparagus filicinus* Ham. ex D. Don | *Asparagus filicinus*Buch.-Ham. ex D.Don | Liliaceae | nye shing | sngo ldum | ldum bu thang sman |
| *Asperugo procumbens* L. | *Asperugo procumbens*L. | Boraginaceae | sgyib ma | sngo ldum | sngo sman |
| *Aster batangensis* Bur. et Franch. | *Aster batangensis*Bureau & Franch. | Asteraceae | ming can chung ba | sngo ldum | sngo sman |
| *Aster flaccidus* Bunge | *Aster flaccidus*Bunge | Asteraceae | lug mig che ba | sngo ldum | sngo sman |
| *Aster himalaicus* C. B. Clarke | *Aster himalaicus*C.B.Clarke | Asteraceae | me tog lug mig | sngo ldum | sngo sman |
| *Aster megalanthus* Ling. | *Aster megalanthus*Y.Ling | Asteraceae | lug mig stong 'khor | sngo ldum | sngo sman |
| *Aster prainii* (Drumm.) Y. L. Chen | *Aster prainii* (J.R.Drumm.) Y.L.Chen | Asteraceae | tshangs pa lha'i me tog | sngo ldum | sngo sman |
| *Aster smithianus* Hand.-Mazz. | *Aster smithianus* Hand.-Mazz. | Asteraceae | yu gu shing nag po | sngo ldum | ldum bu thang sman |
| *Aster tataricus* L. f. | *Aster tataricus*L.f. | Asteraceae | chu de wa | sngo ldum | sngo sman |
| *Aster yunnanensis* Franch. | *Aster yunnanensis*Franch. | Asteraceae | ming can dbang phyug mig | sngo ldum | ldum bu thang sman |
| *Astragalus floridulus* Podlech | *Astragalus floridulus*Podlech | Fabaceae | skam skyes srad dkar | sngo ldum | sngo sman |
| *Astragalus tanguticus* Batalin. | *Astragalus tanguticus*Batalin | Fabaceae | srad sngon | sngo ldum | sngo sman |
| *Astragalus tatsienensis* Bur. et Franch. | *Astragalus tatsienensis Bureau* & Franch. | Fabaceae | srad ser | sngo ldum | sngo sman |
| *Astragalus tongolensis* Ulbr. | *Astragalus tongolensis*Ulbr. | Fabaceae | klu 'dul na po dar ya kan | sngo ldum | sngo sman |
| *Aucklandia lappa* Decne. | *Aucklandia lappa* DC. | Asteraceae | sha pho ru rta | sngo ldum | ldum bu thang sman |
| *Avena sativa* L. | *Avena sativa* L. | Poaceae | yug po | zhing skyes 'bru'i khal | zhing skyes 'bru'i khal |
| *Azadirachta Indica* A.Juss. | Azadirachta indica A.Juss. | Meliaceae | nim pa | shing sman | shing sman |
| *Bambusa tuldoides* Munro | *Bambusa tuldoides*Munro | Poaceae | smyug rtsi | zhing skyes 'bru'i khal | zhing skyes 'bru'i khal |
| *Begonia picta* J.E. Smith | *Begonia picta*Sm. | Begoniaceae | su mi | sngo ldum | shing sman |
| *Berberis jamesiana* Forrest et W. W. Smith | *Berberis jamesiana*Forrest & W.W.Sm. | Berberidaceae | skyer dkar | shing sman | shing sman |
| *Berberis tsarica* Ahrendt | *Berberis tsarica*Ahrendt | Berberidaceae | skyer nag | shing sman | shing sman |
| *Bergenia purpurascens* (Hook f. et Thoms) Engl. | *Bergenia purpurascens*(Hook.f. & Thomson) Engl. | Saxifragaceae | ga dur | sngo ldum | sngo sman |
| *Betula albosinensis* Burk. | *Betula albosinensis*Burkill | Betulaceae | gro ga smug po | shing sman | shing sman |
| *Betula platyphylla* Suk. | *Betula platyphylla*Sukaczev | Betulaceae | gro ga | shing sman | shing sman |
| *Bletilla striata* (Thunb. ex A. Murray) Rchb. f. | *Bletilla striata*(Thunb.) Rchb.f. | Orchidaceae | pa to la | sngo ldum | sngo sman |
| *Boletus edulis* Bull. | *Boletus edulis* Bull. | Boletaceae | glang sha ma | sngo ldum | sngo sman |
| *Bombyx mori* L. | *Bombyx mori L.* | Bombycidae | dar 'bu | sngo ldum | sngo sman |
| *Boschniakia himalaica* Hook. f. et Thoms | *Boschniakia himalaica*Hook.f. & Thomson | Orobanchaceae | ldum so cha | sngo ldum | sngo sman |
| *Brassica campestris* L. | *Brassica campestris* L. | Brassicaceae | pad kha | zhing skyes 'bru'i khal | zhing skyes 'bru'i khal |
| *Brassica juncea* (L.) Czern. et Coss. | *Brassica juncea* (L.) Czern. | Brassicaceae | mar nag | zhing skyes 'bru'i khal | zhing skyes 'bru'i khal |
| *Brassica rapa* L. | *Brassica rapa*L. | Brassicaceae | nyungs ma | zhing skyes 'bru'i khal | zhing skyes 'bru'i khal |
| *Buddleja crispa* Benth. | *Buddleja crispa*Benth. | Loganiaceae | shing a krong | shing sman | shing sman |
| *Bupleurum marginatum* Wall. ex DC. | *Bupleurum marginatum*Wall. ex DC. | Apiaceae | zi ra ser po | sngo ldum | sngo sman |
| *Butea monosperma* (Lam.) Kuntze. | *Butea monosperma*(Lam.) Taub*.* | Fabaceae | ma ru tse | shing sman | shing sman |
| *Caesalpinia crista* Linn. | *Caesalpinia crista*L. | Fabaceae | 'jam 'bras | shing sman | shing sman |
| *Calamus tetradactylus* Hance | *Calamus tetradactylus*Hance | Arecaceae | sba | shing sman | shing sman |
| *Calendula officinalis* L. | *Calendula officinalis*L. | Asteraceae | le brgan chung ba | sngo ldum | sngo sman |
| *Callianthemum pimpinelloides* (D. Don) Hook. f. et Thoms | *Callianthemum pimpinelloides*(D.Don ex Royle) Hook.f. & Thomson | Ranunculaceae | rog po 'joms skyes | sngo ldum | sngo sman |
| *Caltha scaposa* Hook. f. et Thoms. | *Caltha scaposa*Hook.f. & Thomson | Ranunculaceae | rta rming che ba | sngo ldum | sngo sman |
| *Camellia japonica* L. | *Camellia japonica*L. | Theaceae | shan ja | shing sman | shing sman |
| *Camellia sinensis*（L.）O. Ktze | *Camellia sinensis*(L.) Kuntze | Theaceae | ja shing | shing sman | shing sman |
| *Canavalia gladiata* (Jacq.) DC. | *Canavalia gladiata* (Jacq.) DC. | Fabaceae | mkhal zho dmar po | shing sman | shing sman |
| *Cannabis sativa* L. | *Cannabis sativa*L. | Cannabaceae | sro ma nag po | sngo ldum | ldum bu thang sman |
| *Capsella bursa-pastoris* (Linn.) Medic. | *Capsella bursa-pastoris*(L.) Medik. | Brassicaceae | sog ka pa | sngo ldum | sngo sman |
| *Capsicum annuum* L. | *Capsicum annuum*L*.* | Solanaceae | sir pan | shing sman | shing sman |
| *Capsicum frutescens* L. | *Capsicum frutescens* L. | Solanaceae | tsi tra ka | shing sman | shing sman |
| *Caragana brevifolia* Kom. | *Caragana brevifolia*Kom. | Fabaceae | ra tsher | shing sman | shing sman |
| *Caragana changduensis* Liou f. | *Caragana changduensis*Y.X.Liou | Fabaceae | mdzo mo shing | shing sman | shing sman |
| *Caragana erinacea* Kom. | *Caragana erinacea* Kom. | Fabaceae | bra ma | sngo ldum | sngo sman |
| *Caragana jubata* (Pall.) Poir. | *Caragana jubata*(Pall.) Poir. | Fabaceae | mdzo mo shing chung ba | shing sman | shing sman |
| *Cardamine macrophylla* Willd. | *Cardamine macrophylla* Willd. | Brassicaceae | chu rug pa | sngo ldum | sngo sman |
| *Carduus nutans* L. | *Carduus nutans*L. | Asteraceae | spyang tsher rgod pa | sngo ldum | sngo sman |
| *Carthamus tinctorius* L. | *Carthamus tinctorius* L. | Asteraceae | ldum gur gum | rtsi sman | rtsi sman |
| *Carum carvi* L. | *Carum carvi*L. | Apiaceae | go snyod | shing sman | shing sman |
| *Caryopteris trichosphaera* W. W. Sm. | *Caryopteris trichosphaera*W.W.Sm. | Verbenaceae | phur mong a ka ru | sngo ldum | ldum bu thang sman |
| *Cassia fistula* Linn. | *Cassia fistula* L. | Fabaceae | dong ga | shing sman | shing sman |
| *Catabrosa aquatica*（L.）Beauv. | *Catabrosa aquatica*(L.) P.Beauv. | Poaceae | 'dam bu ka ra | sngo ldum | sngo sman |
| *Ceratostigma minus* Stapf ex Prain. | *Ceratostigma minus*Stapf ex Prain | Plumbaginaceae | bya pho tsi | sngo ldum | sngo sman |
| *Chaenomeles speciosa* (Sweet)Nakai | *Chaenomeles speciosa*(Sweet) Nakai | Rubiaceae | bse yab | shing sman | shing sman |
| *Chelonopsis albiflora* Pax et Hoffm. ex Limpr. | *Chelonopsis albiflora* Pax et Hoffm. ex Limpr. | Lamiaceae | ba sha ka dman pa | sngo ldum | ldum bu thang sman |
| *Chenopodium album* L. | *Chenopodium album*L. | Chenopodiaceae | sne'u | sngo ldum | sngo sman |
| *Chenopodium botrys* L. | *Chenopodium botrys* L. | Chenopodiaceae | mon sne | sngo ldum | sngo sman |
| *Choerospondias axillaris* (Roxb.) Burtt. et Hill | *Choerospondias axillaris* (Roxb.) B.L.Burtt & A.W.Hill | Anacardiaceae | snying zho sha | shing sman | shing sman |
| *Chrysanthemum tatsienense* Bur. et Franch. | Chrysanthemum tatsienense Bur. et Franch. | Asteraceae | a byag | sngo ldum | sngo sman |
| *Chrysosplenium carnosum* Hook f. et Thoms | *Chrysosplenium carnosum*Hook.f. & Thomson | Saxifragaceae | g.ya' kyi ma | sngo ldum | sngo sman |
| *Chrysosplenium davidianum* Decne. ex. Maxim. | *Chrysosplenium davidianum*Decne. ex Maxim. | Saxifragaceae | dngul kyi | sngo ldum | sngo sman |
| *Chrysosplenium griffithii* Hook f. et Thoms | *Chrysosplenium griffithii*Hook.f. & Thomson | Saxifragaceae | gseer kyi | sngo ldum | sngo sman |
| *Chrysosplenium nepalense* D. Don. | *Chrysosplenium nepalense*D.Don | Saxifragaceae | zangs kyi | sngo ldum | ldum bu thang sman |
| *Chrysosplenium nudicaule* Bunge | *Chrysosplenium nudicaule*Bunge | Saxifragaceae | lcags kyi | sngo ldum | sngo sman |
| *Cimicifuga foetida* L. | *Cimicifuga foetida* L. | Ranunculaceae | rgya rtsi | sngo ldum | ldum bu thang sman |
| *Cinnamomum camphora* (L.) Persl | *Cinnamomum camphora*(L.) J.Presl | Lauraceae | ga pur shing | rtsi sman | rtsi sman |
| *Cinnamomum cassia* Presl | *Cinnamomum cassia*(L.) J.Presl | Lauraceae | shing tsha | shing sman | shing sman |
| *Cinnamomum glanduliferum* （Wall.）Nees | *Cinnamomum glanduliferum*(Wall.) Meisn. | Lauraceae | ar dmar | shing sman | shing sman |
| *Cinnamomum glanduliferum* （Wall.）Nees | *Cinnamomum glanduliferum*(Wall.) Meisn. | Lauraceae | ar dmar ram go snyod | shing sman | shing sman |
| *Cirsium souliei* (Franch.) Mattf. | *Cirsium souliei*(Franch.) Mattf. ex Rehder & Kobuski | Asteraceae | spyang tsher g.yung ba | sngo ldum | sngo sman |
| *Clematis montana* Buch.-Ham.ex DC. | *Clematis montana*Buch.-Ham. ex DC. | Ranunculaceae | kri shing kri mo | shing sman | shing sman |
| *Clematis rehderiana* Craib | *Clematis rehderiana Craib* | Ranunculaceae | dbyi mong | shing sman | shing sman |
| *Clematis tenuifolia* Royle. | *Clematis tenuifolia* Royle. | Ranunculaceae | dbyi mong nag po | shing sman | shing sman |
| *Cocos nucifera* L. | *Cocos nucifera*L. | Arecaceae | be ta | shing sman | shing sman |
| *Codonopsis canescens* Nannf. | Codonopsis canescens Nannf. | Campanulaceae | klu bdud dkar po | sngo ldum | sngo sman |
| *Codonopsis convolvulacea* Kurz | *Codonopsis convolvulacea* Kurz | Campanulaceae | snyi ba | sngo ldum | ldum bu thang sman |
| *Codonopsis foetens* Hook. f. et Thoms. subsp. *nervosa* (Chipp) D. Y. Hong | *Codonopsis foetens*Hook.f. & Thomson | Campanulaceae | klu bdud rdo rje | sngo ldum | sngo sman |
| *Codonopsis levicalyx* L. T. Shen | *Codonopsis levicalyx*L.D.Shen | Campanulaceae | klu bdud g.yu zhags ma | sngo ldum | sngo sman |
| *Codonopsis thalictrifolia* Wall. | *Codonopsis thalictrifolia*Wall. | Campanulaceae | klu bdud rdo rje sbubs ring | sngo ldum | sngo sman |
| *Commiphora mukul* Engl | *Commiphora mukul*(Hook. ex Stocks) Engl | Burseraceae | gu gul | shing sman | shing sman |
| *Coprinus atramentarius* (Bull. ex Fr.)Fr. | *Coprinus atramentarius* (Bull. ex Fr.) Fr. | PsathYrellaceae | lud kyi sha mong | sngo ldum | sngo sman |
| *Coptis chinensis* Franch. | *Coptis chinensis*Franch. | Ranunculaceae | we len | sngo ldum | sngo sman |
| *Coptis omeiensis*（Chen）C.Y. Cheng | *Coptis omeiensis*(C.Chen) C.Y.Cheng | Ranunculaceae | ye len | sngo ldum | sngo sman |
| *Coptis teeta* Wall. | *Coptis teeta*Wall. | Ranunculaceae | myang rtsi spras | sngo ldum | sngo sman |
| *Corallodiscus flabellatus* (Craib.) Burtt | *Corallodiscus flabellatus* (Craib.) Burtt | Gesneriaceae | brag skya dkar po | sngo ldum | sngo sman |
| *Corallodiscus Kingianus* (Craib) Burtt | *Corallodiscus kingianus*(Craib) B.L.Burtt | Gesneriaceae | brag skya ha bo | sngo ldum | sngo sman |
| *Cordyceps sinensis* (Berk.) Sacc. | *Cordyceps sinensis* (Berk.) Sacc. | Ciavieps | rtswa da byid | sngo ldum | sngo sman |
| *Coriandrum sativum* L. | *Coriandrum sativum*L. | Apiaceae | 'u su | sngo ldum | sngo sman |
| *Corydalis adunca* Maxim. | *Corydalis adunca*Maxim. | Papaveraceae | lug ngal pa | sngo ldum | sngo sman |
| *Corydalis bulbifera* C. Y. Wu | *Corydalis bulbifera*C.Y.Wu | Papaveraceae | de wa mo | sngo ldum | sngo sman |
| *Corydalis chrysosphaera* C. Marquand et Airy Shaw. | *Corydalis chrysosphaera*Marquand & Airy Shaw | Papaveraceae | rtsi dmar rkang gcig | sngo ldum | sngo sman |
| *Corydalis conspersa* Maxim. | *Corydalis conspersa*Maxim. | Papaveraceae | stong ri zil pa | sngo ldum | shing sman |
| *Corydalis crispa* Prain. | *Corydalis crispa*Prain | Papaveraceae | g.yu sdong gser mgo | sngo ldum | sngo sman |
| *Corydalis curviflora* Maxim. | *Corydalis curviflora*Maxim. ex Hemsl. | Papaveraceae | g.yu 'brug zil pa | sngo ldum | shing sman |
| *Corydalis dasyptera* Maxim. | *Corydalis dasyptera*Maxim. | Papaveraceae | rgu drus | sngo ldum | sngo sman |
| *Corydalis edulis* Maxim. | *Corydalis edulis*Maxim. | Papaveraceae | smug chung zil pa | sngo ldum | shing sman |
| *Corydalis hendersonii* Hemsl | *Corydalis hendersonii*Hemsl. | Papaveraceae | rtsi dmar re skon | sngo ldum | sngo sman |
| *Corydalis kingii* Prain | *Corydalis kingii*Prain | Papaveraceae | dom nag zil pa | sngo ldum | shing sman |
| *Corydalis linarioides* Maxim. | *Corydalis linarioides*Maxim. | Papaveraceae | rgya stag zil pa | sngo ldum | shing sman |
| *Corydalis melanochlora* Maxim. | *Corydalis melanochlora*Maxim. | Papaveraceae | de wa | sngo ldum | shing sman |
| *Corydalis mucronifera* Maxim. | *Corydalis mucronifera* Maxim. | Papaveraceae | rtsi dmar zil pa | sngo ldum | sngo sman |
| *Corydalis nigro-apiculata* C. Y. Wu | *Corydalis nigroapiculata* C.Y.Wu | Papaveraceae | seng chung zil pa | sngo ldum | shing sman |
| *Corydalis scaberula* Maxim. | *Corydalis scaberula*Maxim*.* | Papaveraceae | seng ge zil pa | sngo ldum | shing sman |
| *Corydalis wuzhengyiana* Z. Y. Su et Liden | *Corydalis wuzhengyiana*Z.Y.Su & Lidén | Papaveraceae | klung skyes lug ngal | sngo ldum | sngo sman |
| *Corydalis yanhusuo* W. T. Wang ex Z. Y. Su et C. Y. Wu | *Corydalis yanhusuo*(Y.H.Chou & Chun C.Hsu) W.T.Wang ex Z.Y.Su & C.Y.Wu | Papaveraceae | su mi ser po | sngo ldum | shing sman |
| *Cosmos bipinnatus* Cav. | *Cosmos bipinnatus*Cav. | Asteraceae | gangs la (puN+Da ri ka) | sngo ldum | sngo sman |
| *Cotoneaster adpressus* Bois | *Cotoneaster adpressus*Bois | Rubiaceae | tshar leb | shing sman | shing sman |
| *Cotoneaster multiflorus* Bge. var. *atropurpureus* Yu | *Cotoneaster multiflorus*Bunge | Rubiaceae | tshar nag | shing sman | shing sman |
| *Cotoneaster tenuipes* Rehd et Wils. | *Cotoneaster tenuipes*Rehder & E.H.Wilson | Rubiaceae | tshar bu | shing sman | shing sman |
| *Cremanthodium decaisnei* C. B. Clarke. | *Cremanthodium decaisnei*C.B.Clarke | Asteraceae | sha la yu thung | sngo ldum | ldum bu thang sman |
| *Cremanthodium ellisii* (Hook.f.) Kitam. | *Cremanthodium ellisii*(Hook.f.) Kitam. | Asteraceae | sga sho | sngo ldum | ldum bu thang sman |
| *Cremanthodium purpureifolium* Kitam. | *Cremanthodium purpureifolium*Kitam. | Asteraceae | sngo sga | sngo ldum | ldum bu thang sman |
| *Crocus sativus* L. | *Crocus sativus*L. | Iridaceae | gur gum | rtsi sman | rtsi sman |
| *Croton tiglium* L. | *Croton tiglium*L. | Euphorbiaceae | dan rog | shing sman | shing sman |
| *Cuminum cyminum* L. | *Cuminum cyminum* L. | Apiaceae | zi ra dkar po | sngo ldum | sngo sman |
| *Cupressus gigantea* Cheng et L. K. Fu | *Cupressus gigantea*W.C.Cheng & L.K.Fu | Cupressaceae | rong gi shug rgan | shing sman | shing sman |
| *Curcuma longa* L. | *Curcuma longa*L. | Zingiberaceae | yung ba | sngo ldum | ldum bu thang sman |
| *Cuscuta europaea* L. | *Cuscuta europaea*L. | Scrophulariaceae | sbrul zhags pa | sngo ldum | ldum bu thang sman |
| *Cyananthus sherriffii* Cowan | *Cyananthus sherriffii* Cowan | Campanulaceae | sngon bu | sngo ldum | ldum bu thang sman |
| *Cynanchum vincetoxicum* （L.）Pers. | *Cynanchum vincetoxicum* （L.）Pers. | Asclepiadaceae | sngo dug nyung ser po | sngo ldum | sngo sman |
| *Cynoglossum amabile* Stapf et Drumm. | *Cynoglossum amabile*Stapf & J.R.Drumm*.* | Boraginaceae | g.yu lo | sngo ldum | sngo sman |
| *Cynoglossum wallichii* G.Don var. *glochidiatum* (Wall. ex. Benth.) Kazmi | *Cynoglossum wallichii*G.Don | Boraginaceae | 'byar ma | shing sman | shing sman |
| *Cypripedium tibeticum* King ex Rolfe | *Cypripedium tibeticum* King ex Rolfe | Orchidaceae | khu byug pa | sngo ldum | sngo sman |
| *Daphne genkwa* Sieb. et Zucc. | *Daphne genkwa*Siebold & Zucc. | Thymelaeaceae | rngas nag | shing sman | shing sman |
| *Daphne tangutica* Maxim. | *Daphne tangutica*Maxim. | Thymelaeaceae | srin shing sna ma | shing sman | shing sman |
| *Datura stramonium* Linn. | *Datura stramonium*L. | Solanaceae | d+ha du ra | sngo ldum | ldum bu thang sman |
| *Delphinium albocoeruleum* Maxim. | *Delphinium albocoeruleum*Maxim. | Ranunculaceae | lo btsan pa | sngo ldum | sngo sman |
| *Delphinium brunonianum* Royle | *Delphinium brunonianum*Royle | Ranunculaceae | bya rgod spos | sngo ldum | sngo sman |
| *Delphinium caeruleum* Jacq. ex Camb. | *Delphinium caeruleum*Jacquem. ex Cambess. | Ranunculaceae | bya rkang pa | sngo ldum | sngo sman |
| *Delphinium chrysotrichum* Finet et Gagn. | *Delphinium chrysotrichum*Finet & Gagnep. | Ranunculaceae | bya rgod spos chung | sngo ldum | sngo sman |
| *Delphinium gyalanum* Macq. et Shaw | Delphinium gyalanum C.Marquand & Airy Shaw | Ranunculaceae | g.yu lung pa | sngo ldum | sngo sman |
| *Delphinium sherriffii* Munz. | *Delphinium sherriffii*Munz | Ranunculaceae | spyang dug pa | sngo ldum | sngo sman |
| *Delphinium trichophorum* Franch. | *Delphinium trichophorum* Franch. | Ranunculaceae | ga bur tis lo | sngo ldum | sngo sman |
| *Dendrobium nobile* Lindl. | *Dendrobium nobile* Lindl. | Orchidaceae | pu shel tse | sngo ldum | sngo sman |
| *Descurainia sophia* (L.) Webb ex Prantl | *Descurainia sophia*(L.) Webb ex Prantl | Brassicaceae | shang tshe | sngo ldum | sngo sman |
| *Dilophia fontana* Maxim. | *Dilophia fontana* Maxim. | Brassicaceae | byi tsha la phug | sngo ldum | sngo sman |
| *Diospyros kaki* Thunb. | *Diospyros*kaki L.f. | Ebenaceae | rgya yung ba tshwa kha (hri tsi) | shing sman | shing sman |
| *Dipsacus chinensis* Batal | *Dipsacus chinensis*Batalin | Dipsacaceae | spang rtsi 'byar bag can | sngo ldum | sngo sman |
| *Dolomiaea souliei* (Franch.) Shih | *Dolomiaea souliei*(Franch.) C.Shih | Asteraceae | ru rta | sngo ldum | ldum bu thang sman |
| *Dracocephalum bullatum* Forrest ex Diels | *Dracocephalum bullatum*Forrest ex Diels | Lamiaceae | rgod spos | sngo ldum | sngo sman |
| *Dracocephalum heterophyllum* Benth. | *Dracocephalum heterophyllum*Benth. | Lamiaceae | 'jib rtsi dkar po | sngo ldum | sngo sman |
| *Dracocephalum tanguticum* Maxim. | *Dracocephalum tanguticum*Maxim. | Lamiaceae | pri yang ku | sngo ldum | sngo sman |
| *Drynaria baronii* Diels. | *Drynaria baronii* Diels. | Drynariaceae | ldum bu re ral | sngo ldum | ldum bu thang sman |
| *Drynaria propinqua* (Wall. ex Mett.) J. Sm. ex Bedd. | *Drynaria propinqua* (Wall. ex Mett.) Bedd. | Drynariaceae | be ljang re ral | sngo ldum | ldum bu thang sman |
| *Dryobalanops aromatica* C.F.Gaertn. | *Dryobalanops aromatica* C.F.Gaertn. | Dipterocarpaceae | shel ga bur | shing sman | shing sman |
| *Elaeocarpus ganitrus* Roxb. | *Elaeocarpus ganitrus* Roxb. | Ericaceae | rag sha | shing sman | shing sman |
| *Elettaria cardamomum* (L.) Maton | *Elettaria cardamomum*(L.) Maton | Zingiberaceae | sug smel chung ba | rtsi sman | rtsi sman |
| *Eleusine coracana* (L.)Gaertn. | *Eleusine coracana* (L.) Gaertn. i | Poaceae | khra ma | zhing skyes 'bru'i khal | zhing skyes 'bru'i khal |
| *Elsholtzia argyi* Lévl. | *Elsholtzia argyi* H.Lév. | Lamiaceae | byi rug sngon po | sngo ldum | sngo sman |
| *Elsholtzia densa* Benth. | *Elsholtzia densa* Benth. | Lamiaceae | byi rug smug po | sngo ldum | sngo sman |
| *Elsholtzia eriostachya* (Benth.) Benth. | *Elsholtzia eriostachya*(Benth.) Benth. | Lamiaceae | byi rug ser po | sngo ldum | sngo sman |
| *Elsholtzia fruticosa*（D. Don）Rehd. | *Elsholtzia fruticosa (D.Don)* Rehder | Lamiaceae | phur smug | sngo ldum | ldum bu thang sman |
| *Embelia laeta* (L.) Mez | *Embelia laeta*(L.) Mez | Myrsinaceae | byi tang ka | shing sman | sngo sman |
| *Entada phaseoloides* (Linn.) Merr. | *Entada phaseoloides*(L.) Merr. | Fabaceae | mchin pa zho sha | shing sman | shing sman |
| *Ephedra intermedia* Schrenk ex Mey. | *Ephedra intermedia*Schrenk & C.A.Mey. | Ephedraceae | mtshe ldum | sngo ldum | ldum bu thang sman |
| *Ephedra likiangensis* Florin f. mairei (Florin) C. Y. Cheng. | *Ephedra likiangensis*Florin | Ephedraceae | spang mtshe | sngo ldum | ldum bu thang sman |
| *Ephedra saxatilis* Royle ex Florin. | *Ephedra saxatilis* Royle ex Florin. | Ephedraceae | spang mtshe | sngo ldum | ldum bu thang sman |
| *Epilobium angustifolium* L. | *Epilobium angustifolium*L. | Onagraceae | sngo dug mo nyung dmar po | sngo ldum | sngo sman |
| *Epilobium cylindricum* D. Don | *Epilobium cylindricum*D.Don | Onagraceae | sngo dug mo nyung | sngo ldum | sngo sman |
| *Equisetum arvense* L. | *Equisetum arvense*L. | Equisetaceae | chu mtshe | sngo ldum | ldum bu thang sman |
| *Eriophyton wallichii* Benth. | *Eriophyton wallichii*Benth. | Lamiaceae | shing len smug po | sngo ldum | sngo sman |
| *Eritrichium sinomicrocarpum* W.T.Wang. | *Eritrichium sinomicrocarpum*W.T.Wang | Boraginaceae | g.yu chung ba | sngo ldum | sngo sman |
| *Erodium tibetanum* Edgew. | *Erodium tibetanum*Edgew. & Hook.f. | Geraniaceae | spor chung gab skyes | sngo ldum | sngo sman |
| *Erysimum chamaephyton* Maxim. | *Erysimum chamaephyton* Maxim. | Brassicaceae | zangs tig rma bya chig thub | sngo ldum | ldum bu thang sman |
| *Erysimum hieraciifolium* L. | *Erysimum hieraciifolium*L. f. | Brassicaceae | mu nyungs ser po | sngo ldum | sngo sman |
| *Erysimum longisiliquum* Hook f. et Thoms | *Erysimum longisiliquum* Hook f. et Thoms | Brassicaceae | gser tig | sngo ldum | ldum bu thang sman |
| *Eugenia caryophllata* Thunb | *Eugenia caryophllata Thunb* | Myrtaceae | li shi | shing sman | shing sman |
| *Euonymus sanguineus* Loes | *Euonymus sanguineus*Loes. ex Diels | Celastraceae | ma bzhog gru bzhi | shing sman | shing sman |
| *Euphorbia fischeriana* Steud. | *Euphorbia fischeriana*Steud. | Euphorbiaceae | dur byid | sngo ldum | ldum bu thang sman |
| *Euphorbia micractina* Boiss | *Euphorbia micractina*Boiss. | Euphorbiaceae | khron bu | sngo ldum | ldum bu thang sman |
| *Euphorbia stracheyi* Boiss | *Euphorbia stracheyi*Boiss. | Euphorbiaceae | khron bu chung ba | sngo ldum | ldum bu thang sman |
| *Euphorbia wallichii* Hook. f. | *Euphorbia wallichii*Hook.f. | Euphorbiaceae | thar nu | sngo ldum | ldum bu thang sman |
| *Fagopyrum dibotrys* (D. Don) Hara. | *Fagopyrum dibotrys* (D. Don) Hara. | Polygonaceae | bra rgod | sngo ldum | sngo ldum |
| *Fagopyrum esculentum* Moench | *Fagopyrum esculentum*Moench | Polygonaceae | bra bo | sngo ldum | sngo sman |
| *Ferula assafoetida* L. | *Ferula assa-foetida* L. | Apiaceae | shing kun | sngo ldum | sngo sman |
| *Foeniculum vulgare* Mill. | *Foeniculum*vulgare Mill. | Apiaceae | la la phud | sngo ldum | sngo sman |
| *Fragaria orientalis* Lozinsk. | *Fragaria orientalis*Losinsk. | Rubiaceae | tsi ta sa 'dzin | sngo ldum | sngo sman |
| *Fraxinus rhynchophylla* Hance | *Fraxinus rhynchophylla* Hance | Oleaceae | stab seng | shing sman | shing sman |
| *Fritillaria cirrhosa* D.Don | *Fritillaria cirrhosa*D.Don | Liliaceae | a'u rtsi | sngo ldum | sngo sman |
| *Fritillaria delavayi* Franch. | *Fritillaria delavayi*Franch. | Liliaceae | a bi Sha | sngo ldum | sngo sman |
| *Lloydia serotina* (L.) Rchb. var. *parva* (Marq.et Shaw) Hara. | *Gagea serotina* (L.) Ker Gawl. | Liliaceae | a 'dra | sngo ldum | sngo sman |
| *Galeopsis bifida* Boenn | *Galeopsis bifida*Boenn. | Lamiaceae | zhim thig sangs rgyas chu 'jip | sngo ldum | sngo sman |
| *Galium aparine* Linn. | *Galium aparine*L. | Rubiaceae | zangs rtsi dkar po | sngo ldum | sngo sman |
| *Gastrodia elata* Bl. | *Gastrodia elata*Blume | Orchidaceae | ra mo shag chen | sngo ldum | sngo sman |
| *Gentiana algida* Pall. | *Gentiana algida*Pall. | Gentianaceae | spang rgyan dkar po 'bring ba | sngo ldum | sngo sman |
| *Gentiana crassicaulis* Duthie ex Burk. | *Gentiana crassicaulis*Duthie ex Burkill | Gentianaceae | kyi lce nag po | sngo ldum | sngo sman |
| *Gentiana erectosepala* T. N. Ho. | *Gentiana erectosepala* T. N. Ho. | Gentianaceae | spang rgyan dkar po chung ba rnam gcig | sngo ldum | sngo sman |
| *Gentiana lawrencei* Burkill var. *farreri* (I. B. Balfour) T. N. Ho. | *Gentiana lawrencei*Burkill | Gentianaceae | spang rgyan sngon po | sngo ldum | sngo sman |
| *Gentiana lhassica* Burk. | *Gentiana lhassica*Burkill | Gentianaceae | spang rgyan sngo chung | sngo ldum | sngo sman |
| *Gentiana nubigena* Edgew. | *Gentiana nubigena*Edgew. | Gentianaceae | spang rgyan dkar po chung ba rnam gcig | sngo ldum | sngo sman |
| *Gentiana obconica* T. N. Ho | *Gentiana obconica*T. N. Ho | Gentianaceae | spang rgyan nag chung | sngo ldum | sngo sman |
| *Gentiana robusta* King ex Hook . f. | *Gentiana robusta*King ex Hook.f. | Gentianaceae | kyi lce dkar po che ba | sngo ldum | sngo sman |
| *Gentiana stipitata* Edgew. | *Gentiana stipitata*Edgew. | Gentianaceae | spang rgyan sngon po 'bring ba | sngo ldum | sngo sman |
| *Gentiana straminea* Maxim. | *Gentiana straminea*Maxim. | Gentianaceae | kyi lce dkar po | sngo ldum | sngo sman |
| *Gentiana szechenyii* Kanitz | *Gentiana szechenyii*Kanitz | Gentianaceae | spang rgyan dkar po | sngo ldum | sngo sman |
| *Gentiana tibetica* King ex Hook. f. | *Gentiana tibetica*King ex Hook.f. | Gentianaceae | kyi lce nag po che ba | sngo ldum | sngo sman |
| *Gentiana urnula* H. Smith. | *Gentiana urnula*Harry Sm. | Gentianaceae | gang gA chung | sngo ldum | sngo sman |
| *Gentiana veitchiorum* Hemsl. | *Gentiana veitchiorum*Hemsl. | Gentianaceae | spang rgyan nag po | sngo ldum | ldum bu thang sman |
| *Gentiana waltonii* Burk. | *Gentiana waltonii*Burkill | Gentianaceae | kyi lce nag po chung ba | sngo ldum | sngo sman |
| *Gentianopsis grandis*（H. Smith）Ma. | *Gentianopsis grandis* (Harry Sm.) Ma | Gentianaceae | lcags ting dkar po | sngo ldum | sngo sman |
| *Geranium pratense* L. | *Geranium pratense* L. | Geraniaceae | spor chen | sngo ldum | sngo sman |
| *Geranium pylzowianum* Maxim. | *Geranium pylzowianum*Maxim. | Geraniaceae | gla sgang g.yung ba | sngo ldum | sngo sman |
| *Geranium refractum* Edgew. et. Hook. f. | *Geranium refractum*Edgew. & Hook.f. | Geraniaceae | spor chung | sngo ldum | sngo sman |
| *Glycine max* (Linn.)Merr. | *Glycine max*(L.) Merr. | Fabaceae | sran chen dkar po | zhing skyes 'bru'i khal | zhing skyes 'bru'i khal |
| *Glycyrrhiza uralensis* Fisch. | *Glycyrrhiza uralensis*Fisch. | Fabaceae | shing mngar | shing sman | shing sman |
| *Gnaphalium affine* D.Don. | *Gnaphalium affine* D.Don. | Asteraceae | gan+d+ha b+ha dra | sngo ldum | sngo sman |
| *Gnaphalium hypoleucum* DC. | *Gnaphalium hypoleucum* DC. | Asteraceae | gan+d+ha b+ha dra ser po | sngo ldum | sngo sman |
| *Gossampinus malabarica* (DC.)Merr. | *Gossampinus malabarica* (DC.) Merr. | Bombacaceae | nA ga ge sar | shing sman | shing sman |
| *Gossypium herbaceum* L. | *Gossypium herbaceum*L. | Malvaceae | ras 'bras | shing sman | shing sman |
| *Gymnadenia orchidis* Lindl. | *Gymnadenia orchidis*Lindl. | Orchidaceae | dbang po lag pa | sngo ldum | sngo sman |
| *Halenia elliptica* D. Don. | *Halenia elliptica*D.Don | Gentianaceae | lcags tig | sngo ldum | ldum bu thang sman |
| *Halerpestes cymbalaria* (Pursh) Green | *Halerpestes cymbalaria* (Pursh) Greene | Ranunculaceae | gsor ldem pa | sngo ldum | sngo sman |
| *Hedera nepalensis* K. Koch var. *sinensis* (Tobl.) Rehd. | *Hedera nepalensis*K.Koch | Araliaceae | 'brug shing | shing sman | shing sman |
| *Hedysarum sikkimense* Benth. ex Baker. | *Hedysarum sikkimense* Baker | Fabaceae | srad dmar | sngo ldum | sngo sman |
| *Hedysarum tibeticum* (Bentham) B. H. Choi & H. Ohashi | *Hedysarum tibeticum*(Benth.) B.H. Choi & H. Ohashi | Fabaceae | srad smug a lag ta | sngo ldum | sngo sman |
| *Heracleum candicans* Wall. ex DC. | *Heracleum candicans*Wall. ex DC. | Apiaceae | spru dkar | sngo ldum | sngo sman |
| *Heracleum millefolium* Diels | *Heracleum millefolium*Diels | Apiaceae | spru skya | sngo ldum | sngo sman |
| *Herminium monorchis*（L.）R. Br. | *Herminium monorchis*(L.) R.Br. | Orchidaceae | bye lce lag pa | sngo ldum | sngo sman |
| *Herpetospermum pedunculosum* (Ser.) C. B. Clarke | *Herpetospermum pedunculosum* (Ser.) C.B. Clarke | Cucurbitaceae | gser gyi me tog | sngo ldum | sngo sman |
| *Heteropappus crenatifolius*（Hand.-Mzz.）Griers | *Heteropappus crenatifolius* (Hand.-Mazz.) Grierson | Asteraceae | lug chung | sngo ldum | sngo sman |
| *Hippophae rhamnoides* L. | *Hippophae rhamnoides* L. | Elaeagnaceae | star bu | shing sman | shing sman |
| *Hippophae rhamnoides* L. subsp. *gyantsensis* Rousi | *Hippophae rhamnoides* L. subsp. *gyantsensis* Rousi | Elaeagnaceae | gla ba shing | shing sman | shing sman |
| *Hippophae rhamnoides* L. subsp. *turkestanica* Rousi | *Hippophae rhamnoides* L. subsp. *turkestanica* Rousi | Elaeagnaceae | mnga' ris star bu | shing sman | shing sman |
| *Hippophae rhamnoides* L. subsp. *yunnanensis* Rousi | *Hippophae rhamnoides* L. subsp. *yunnanensis* Rousi | Elaeagnaceae | gnam star rnam gcig | shing sman | shing sman |
| *Hippophae tibetana*Schltdl. | *Hippophae tibetana*Schltdl. | Elaeagnaceae | sa star | shing sman | shing sman |
| *Hippuris vulgaris* L. | *Hippuris vulgaris*L. | Hippuridaceae | na shug sdong dmar | sngo ldum | sngo sman |
| *Holarrhena pubescens* Wallich ex G. Don | *Holarrhena pubescens*Wall. ex G.Don | Apocynaceae | dug mo nyung | shing sman | shing sman |
| *Hordeum vulgare* L. | *Hordeum vulgare*L. | Poaceae | so ba | zhing skyes 'bru'i khal | zhing skyes 'bru'i khal |
| *Hordeum vulgare* var. nudum Hook.f. | *Hordeum vulgare*L. | Poaceae | nas | zhing skyes 'bru'i khal | zhing skyes 'bru'i khal |
| *Hornstedtia tibetica* T. L. Wu et S. J. Chen | *Hornstedtia tibetica*T.L.Wu & S.J.Chen | Zingiberaceae | bod sug | rtsi sman | rtsi sman |
| *Hyoscyamus niger* L. | *Hyoscyamus niger*L. | Solanaceae | lang thang tse | sngo ldum | ldum bu thang sman |
| *Hypecoum leptocarpum* Hook. f. et Thoms | *Hypecoum leptocarpum*Hook. f. & Thomson | Papaveraceae | par pa ta | sngo ldum | shing sman |
| *Impatiens sulcata* Wall. | *Impatiens sulcata*Wall. | Balsaminaceae | kan da b+ha ra | sngo ldum | sngo sman |
| *Incarvillea compacta* Maxim. | *Incarvillea compacta*Maxim. | Bignoniaceae | ug chos | sngo ldum | sngo sman |
| *Incarvillea lutea* Bur. et Franch. | *Incarvillea lutea*Bureau & Franch. | Bignoniaceae | ug chos dkar po | sngo ldum | sngo sman |
| *Inula helenium* L. Hook. f. | *Inula helenium*L. | Asteraceae | ma nu pa tra | sngo ldum | ldum bu thang sman |
| *Inula racemosa* Hook. f. | *Inula racemosa*Hook.f. | Asteraceae | push+kar mu la | sngo ldum | ldum bu thang sman |
| *Iris clarkei* Baker | *Iris clarkei*Baker ex Hook.f. | Iridaceae | mon skyes gres ma | sngo ldum | sngo sman |
| *Iris goniocarpa* Baker | *Iris goniocarpa*Baker | Iridaceae | ma ning gres ma | sngo ldum | sngo sman |
| *Iris lactea* Pall. | *Iris lactea*Pall. | Iridaceae | pho gres | sngo ldum | sngo sman |
| *Iris loczyi* Kanitz. | *Iris loczyi*Kanitz | Iridaceae | gres ma | sngo ldum | sngo sman |
| *Iris potaninii* Maxim. | *Iris potaninii*Maxim. | Iridaceae | ko tha zla bsil | sngo ldum | sngo sman |
| *Isodon rubescens* (Hemsley) H. Hara | *Isodon rubescens*(Hemsl.) H.Hara | Lamiaceae | yog mo | sngo ldum | ldum bu thang sman |
| *Ixeris gracilis* DC. Stebb. | *Ixeris gracilis* DC. Stebb. | Asteraceae | rtsa mkhris | sngo ldum | sngo sman |
| *Juglans regia* L. | *Juglans regia*L. | Juglandaceae | star ka | shing sman | shing sman |
| *Juncus leucomelas* Royle ex D.Don | *Juncus leucomelas*Royle ex D.Don | Juncaceae | byi shang dkar mo | sngo ldum | sngo sman |
| *Juniperus formosana* Hayata | *Juniperus formosana*Hayata i | Cupressaceae | shug tsher | shing sman | shing sman |
| *Juniperus pingii* W. C. Cheng ex Ferre var. *wilsonii* (Rehder) Silba. | *Juniperus pingii*W.C.Cheng ex Ferré | Cupressaceae | spa ma | shing sman | shing sman |
| *Juniperus squamata* Buchanan-Hamilton ex D. Don. | *Juniperus squamata*Buch.-Ham. ex D.Don | Cupressaceae | shug pa | shing sman | shing sman |
| *Kaempferia galanga* L. | *Kaempferia galanga*L. | Zingiberaceae | sga 'dra | shing sman | shing sman |
| *Lablab purpureus* (L.) Sweet. | *Lablab purpureus*(L.) Sweet | Fabaceae | mon sran leb mo dkar po | zhing skyes 'bru'i khal | zhing skyes 'bru'i khal |
| *Lactarius deliciosus* (L.ex Fr.) Gray. | *Lactarius deliciosus* (L.ex Fr.) Gray. | Russulaceae | klung gi sha mong | sngo ldum | sngo sman |
| *Lagenaria siceraria* (Molina) Standl. | *Lagenaria siceraria*(Molina) Standl. | Cucurbitaceae | ka ped | shing sman | shing sman |
| *Lagopsis supina* (Steph. ex Willd.) Ik.-Gal. | *Lagopsis supina*(Steph. ex Willd.) Ikonn.-Gal. | Lamiaceae | zhim thig dkar po | sngo ldum | sngo sman |
| *Lagotis brachystachya* Maxim. | *Lagotis brachystachya*Maxim. | Scrophulariaceae | 'bri ta sa 'dzin | sngo ldum | sngo sman |
| *Lagotis ramalana* Batalin | *Lagotis ramalana*Batalin | Scrophulariaceae | hong len pho | sngo ldum | sngo sman |
| *Lagotis yunnanensis* W. W. Smith. | *Lagotis yunnanensis*W.W. Sm. | Scrophulariaceae | hong len | sngo ldum | sngo sman |
| *Lamiophlomis rotata* (Benth. ex Hook. f.) Kudo | *Lamiophlomis rotata* (Benth. ex Hook. f.) Kudo | Lamiaceae | rta lpags | sngo ldum | sngo sman |
| *Lamium amplexicaule* L. | *Lamium amplexicaule*L. | Lamiaceae | zhim thig dmar po | sngo ldum | sngo sman |
| *Lancea tibetica* Hook. f. et Hsuan | *Lancea tibetica*Hook. f. & Thomson | Scrophulariaceae | pa yag pa | sngo ldum | sngo sman |
| *Lasiosphaera fenzii* Reich． | *Lasiosphaera fenzii* Reich． | Lycoperdaceae | 'dre sha ma (pha wang sgo ti) | sngo ldum | ldum bu thang sman |
| *Leibnitzia nepalensis*（Kunze）Kitamura. | *Leibnitzia nepalensis* (Kunze) Kitam. | Asteraceae | khrog chung ba | sngo ldum | sngo sman |
| *Lens culinaris* Medic. | *Lens culinaris* Medik. | Fabaceae | sran chung leb mo | shing sman | shing sman |
| *Leontopodium franchetii* Beauv. | *Leontopodium franchetii*Beauverd | Asteraceae | spra thog pa | sngo ldum | sngo sman |
| *Lepidium apetalum* Willd. | *Lepidium apetalum* Willd. | Brassicaceae | dar ya kan | sngo ldum | sngo sman |
| *Lepisorus soulieanus* (Christ) Ching et S. K. Wu | *Lepisorus soulieanus*(Christ) Ching & S.K. Wu | Polypodiaceae | brag spos | sngo ldum | sngo sman |
| *Lethariella cladonioides* (Nyl.) Krog | *Lethariella cladonioides* (Nyl.) Krog | parmeliaceae | spang tshan gser skud | sngo ldum | sngo sman |
| *Ligularia lapathifolia*（Franch.）Hand.-Mazz. | *Ligularia lapathifolia (Franch.)* Hand.-Mazz. | Asteraceae | ri sho kro ti | sngo ldum | ldum bu thang sman |
| *Ligularia pleurocaulis* (Franch.) Hand.-Mazz. | *Ligularia pleurocaulis*(Franch.) Hand.-Mazz. | Asteraceae | sha la yu ring | sngo ldum | ldum bu thang sman |
| *Ligularia virgaurea* (Maxim.) Mattf. | *Ligularia virgaurea*(Maxim.) Mattf. ex Rehder & Kobuski | Asteraceae | ri sho | sngo ldum | ldum bu thang sman |
| *Ligusticum pteridophyllum* Franch. | *Ligusticum pteridophyllum*Franch. | Apiaceae | 'bam po | sngo ldum | ldum bu thang sman |
| *Linum pallescens* Bunge | *Linum pallescens*Bunge | Linaceae | zar rgod | zhing skyes 'bru'i khal | zhing skyes 'bru'i khal |
| *Linum usitatissimum* L. | *Linum usitatissimum*L. | Linaceae | zar ma | zhing skyes 'bru'i khal | zhing skyes 'bru'i khal |
| *Litsea cubeba* (Lour.) Pers | *Litsea cubeba*(Lour.) Pers | Lauraceae | snyag | shing sman | shing sman |
| *Lloydia serotina* (L.) Rchb. | *Lloydia serotina* (L.) Rchb. | Liliaceae | a wa | sngo ldum | sngo sman |
| *Lonicera japonica* Thunb. | *Lonicera japonica*Thunb. | Caprifoliaceae | 'phang ma nag po | shing sman | shing sman |
| *Lonicera maackii* (Rupr.) Maxim. | *Lonicera maackii (Rupr.)* Maxim. | Caprifoliaceae | 'phang ma | shing sman | shing sman |
| *Lonicera rupicola* Hook. f. et Thoms. | *Lonicera rupicola*Hook. f. & Thomson | Caprifoliaceae | khyi shing | shing sman | shing sman |
| *Lonicera saccata* Rehd. | *Lonicera saccata* Rehd. | Caprifoliaceae | 'phang skya | shing sman | shing sman |
| *Luffa aegyptiaca* Miller. | *Luffa aegyptiaca* Miller. | Cucurbitaceae | gser gyi phud bu | sngo ldum | sngo sman |
| *Malus pumila* Mill. | *Malus pumila*Mill. | Rubiaceae | ku shu | shing sman | shing sman |
| *Malus rockii* Rehd | *Malus rockii*Rehder | Rubiaceae | skrag pa shing | shing sman | shing sman |
| *Malus toringoides* (Rehd.) Hughes | *Malus toringoides (*Rehder) Hughes | Rubiaceae | 'o se | shing sman | shing sman |
| *Malva cathayensis* M. G. Gilbert, Y. Tang & Dorr | *Malva cathayensis*M.G. Gilbert, Y. Tang & Dorr | Malvaceae | mo lcam | sngo ldum | ldum bu thang sman |
| *Malva verticillata* Linn. | *Malva verticillata*L. | Malvaceae | lcam pa | sngo ldum | ldum bu thang sman |
| *Mandragora chinghaiensis* Kuang et A. M. Lu | *Mandragora* *chinghaiensis* Kuang et A. M. Lu | Solanaceae | kha shog pa | sngo ldum | ldum bu thang sman |
| *Mangifera indica* L. | *Mangifera indica*L. | Anacardiaceae | a 'bras | shing sman | shing sman |
| *Marmoritis complanatum* (Dunn) A. L. Budantzev | *Marmoritis complanata*(Dunn) A.L.Budantzev | Lamiaceae | gnyan 'dul pa | sngo ldum | sngo sman |
| *Meconopsis argemonantha* Prain | *Meconopsis argemonantha*Prain | Papaveraceae | ut+pal dkar po rnam gcig | sngo ldum | sngo sman |
| *Meconopsis betonicifolia* Franch. | *Meconopsis betonicifolia*Franch. | Papaveraceae | ut+pal sga rab | sngo ldum | sngo sman |
| *Meconopsis henrici* Bur et Franch. | *Meconopsis henrici*Bureau & Franch. | Papaveraceae | smug chung 'dan yon | sngo ldum | sngo sman |
| *Meconopsis horridula* Hook. f. et Thoms. | *Meconopsis horridula*Hook. f. & Thomson | Papaveraceae | tsher sngon | sngo ldum | sngo sman |
| *Meconopsis integrifolia*（Maxim.）French. | *Meconopsis integrifolia* (Maxim.) Franch. | Papaveraceae | ut+pal ser po | sngo ldum | sngo sman |
| *Meconopsis paniculata* (D. Don) Prain | *Meconopsis paniculata* (D. Don) Prain | Papaveraceae | ut+pal dkar po | sngo ldum | sngo sman |
| *Meconopsis pseudohorridula* C. Y. Wu. et H. Chuang | *Meconopsis pseudohorridula*C.Y. Wu & H. Chuang | Papaveraceae | tsher dar ya kan | sngo ldum | sngo sman |
| *Meconopsis punicea* Maxim. | *Meconopsis punicea*Maxim. | Papaveraceae | ut+pal dmar po | sngo ldum | shing sman |
| *Meconopsis quintuplinervia* Regel | *Meconopsis quintuplinervia*Regel | Papaveraceae | ut+pal ske ldem | sngo ldum | sngo sman |
| *Meconopsis racemosa Maxim.* | *Meconopsis racemosa*Maxim. | Papaveraceae | tsher sngon rgod pa | sngo ldum | sngo sman |
| *Meconopsis simplicifolia* (D. Don) Walp | *Meconopsis simplicifolia*(D. Don) Walp. | Papaveraceae | ut+pal yu ring | sngo ldum | sngo sman |
| *Meconopsis torquata* Prain | *Meconopsis torquata*Prain | Papaveraceae | ut+pal bdud rtsi 'khyil | sngo ldum | sngo sman |
| Medicago lupulina L. | *Medicago lupulina* L. | Fabaceae | 'bu su hang pho | sngo ldum | sngo sman |
| *Medicago* ruthenica L. | *Medicago ruthenica*(L.) Ledeb. | Fabaceae | 'bu su hang | sngo ldum | sngo sman |
| *Melilotus suaveolens* Ledeb. | *Melilotus suaveolens* Ledeb. | Fabaceae | rgya spos dman pa | sngo ldum | sngo sman |
| *Metroxylon sagu* Rottb. | *Metroxylon sagu*Rottb. | Arecaceae | gram shing | shing sman | shing sman |
| *Microula tibetica* Benth. | *Microula tibetica*Benth. | Boraginaceae | rmun bu | sngo ldum | sngo sman |
| *Millettia pachycarpa* Benth. | *Millettia pachycarpa*Benth. | Fabaceae | khrung then tsi | shing sman | shing sman |
| *Mirabilis himalaica* (Edgew.) Heim. | *Mirabilis himalaica* (Edgew.) Heim. | Nyctaginaceae | a shwa gan+d+ha | sngo ldum | ldum bu thang sman |
| *Momordica cochinchinensis* (Lour.) Spreng. | *Momordica cochinchinensis*(Lour.) Spreng. | Cucurbitaceae | gser me che ba | sngo ldum | sngo sman |
| *Morina kokonorica* Hao | *Morina kokonorica*K.S. Hao | Dipsacaceae | spyang tsher | sngo ldum | sngo sman |
| *Morus alba* L. | *Morus alba*L. | Moraceae | dar shing | shing sman | shing sman |
| *Mucuna sempervirens* Hemsl. | *Mucuna sempervirens*Hemsl. | Fabaceae | gla gor zho sha | shing sman | shing sman |
| *Myricaria germanica* (L.) Desv. | *Myricaria germanica*(L.) Desv. | Tamaricaceae | 'om bu | shing sman | shing sman |
| *Myricaria squamosa* Desv. | *Myricaria squamosa Desv.* | Tamaricaceae | 'om chung | shing sman | shing sman |
| *Myristica fragrans* Houtt． | *Myristica fragrans*Houtt. | Myristicaceae | dza ti | rtsi sman | rtsi sman |
| *Nardostachys jatamansi*（D. Don）DC. | *Nardostachys jatamansi*(D.Don) DC. | Valerianaceae | spang spos | sngo ldum | sngo sman |
| *Nelumbo nucifera* Gaertn. | *Nelumbo nucifera*Gaertn. | Nymphaeaceae | pad rtsa | sngo ldum | sngo sman |
| *Nelumbo nucifera* Gaertn. | *Nelumbo nucifera*Gaertn. | Nymphaeaceae | pad+ma ge sar | sngo ldum | sngo sman |
| *Nepeta coerulescens* Maxim | *Nepeta coerulescens*Maxim. | Lamiaceae | zhim thig sngon po | sngo ldum | sngo sman |
| *Nepeta densiflora* Kar.et Kir. | *Nepeta densiflora*Kar. & Kir. | Lamiaceae | zhim thig sngo chung | sngo ldum | sngo sman |
| *Nepeta dentata* C. Y. Wu et Hsuan | *Nepeta dentata*C.Y.Wu & S.J.Hsuan | Lamiaceae | ma ma 'jib 'jib | sngo ldum | sngo sman |
| *Nepeta angustifolia* C.Y.Wu | *Nepeta hemsleyana*Oliv. ex Prain | Lamiaceae | gza' bdug nag po | sngo ldum | sngo sman |
| *Nigella glandulifera* Freyn et Sint. | *Nigella glandulifera* Freyn et Sint. | Ranunculaceae | zi ra nag po | sngo ldum | sngo sman |
| *Notopterygium forbesii* de Boiss. | *Notopterygium forbesii* de Boiss. | Apiaceae | spru nag | sngo ldum | sngo sman |
| *Notopterygium incisum* Ting ex H. T. Chang | *Notopterygium incisum* K.C.Ting ex H.T.Chang | Apiaceae | spru ser | sngo ldum | sngo sman |
| *Onosma hookeri* Clarke var. *longiflorum* Duthie ex Stapf | *Onosma hookeri*C.B. Clarke | Boraginaceae | 'bri mog | sngo ldum | shing sman |
| *Onosma waddellii* Duthie | *Onosma waddellii*Duthie | Boraginaceae | byis mog | sngo ldum | sngo sman |
| *Orchis latifolia* L. | *Orchis latifolia* L. | Orchidaceae | dbang lag dmar po | sngo ldum | sngo sman |
| *Oreosolen wattii* Hook. f. | *Oreosolen wattii*Hook. f. | Scrophulariaceae | rta lpags dkar po | sngo ldum | sngo sman |
| *Oroxylum indicum* (L.) Bentham ex Kurz | *Oroxylum indicum* (L.) Kurz | Bignoniaceae | tsam pa ka | shing sman | shing sman |
| *Oryza sativa* L. | *Oryza sativa*L. | Poaceae | 'bras | zhing skyes 'bru'i khal | zhing skyes 'bru'i khal |
| *Oxygraphis glacialis* (Fisch. ex DC.) Bunge | *Oxygraphis glacialis* (Fisch. ex DC.) Bunge | Ranunculaceae | dgo ba me tong | sngo ldum | sngo sman |
| *Oxytropis kansuensis* Bunge | *Oxytropis kansuensis*Bunge | Fabaceae | g.yu thog srad dkar | sngo ldum | sngo sman |
| *Oxytropis microphylla* （Pall.） DC. | *Oxytropis microphylla*(Pall.) DC. | Fabaceae | stag sha nag po | sngo ldum | sngo sman |
| *Oxytropis microphylla* (Pall.) DC*.* | *Oxytropis microphylla* (Pall.) DC. | Fabaceae | sngo stag sha | sngo ldum | sngo sman |
| *Oxytropis ochrocephala* Bunge | *Oxytropis ochrocephala*Bunge | Fabaceae | dug srad | sngo ldum | sngo sman |
| *Oxytropis sericopetala* Prain ex C. E. C. Fisch. | *Oxytropis sericopetala*C.E.C.Fisch. | Fabaceae | bye ma rgya skyegs | sngo ldum | sngo sman |
| *Oxytropis squammulosa* DC. | *Oxytropis squammulosa*DC. | Fabaceae | stag sha | sngo ldum | sngo sman |
| *Oxytropis subpodoloba* P. C. Li | *Oxytropis subpodoloba*P.C.Li | Fabaceae | srad nag | sngo ldum | sngo sman |
| *Paeonia veitchii* Lynch. | *Paeonia veitchii* Lynch | Ranunculaceae | ra dug dmar po | sngo ldum | sngo sman |
| *Panax ginseng* C. A. Mey. | *Panax ginseng*C.A.Mey. | Araliaceae | dkar po chig thub | sngo ldum | sngo ldum |
| *Panax notoginseng* (Burkill) F. H. Chen ex C. H. Chow et W. G. Huang | *Panax notoginseng* (Burkill) F.H.Chen | Araliaceae | glang chen chig thub | sngo ldum | sngo ldum |
| *Panicum miliaceum* L. | *Panicum miliaceum*L. | Poaceae | tsi tsi | zhing skyes 'bru'i khal | zhing skyes 'bru'i khal |
| *Papaver rhoeas* L. | *Papaver rhoeas*L. | Papaveraceae | rgya men | sngo ldum | sngo sman |
| *Paraquilegia microphylla* (Royle) Drumm. et Hutch. | *Paraquilegia microphylla*(Royle) J.R. Drumm. & Hutch. | Ranunculaceae | yu mo mde'u 'byin | sngo ldum | sngo sman |
| *Parnassia trinervis* Drude | *Parnassia trinervis* Drude | Saxifragaceae | dngul tig dkar po | sngo ldum | ldum bu thang sman |
| *Pedicularis cranolopha* Maxim. | *Pedicularis cranolopha* Maxim. | Scrophulariaceae | lug ru dkar po | sngo ldum | sngo sman |
| *Pedicularis davidii* Franch. | *Pedicularis davidii*Franch. | Scrophulariaceae | lug ru smug po | sngo ldum | sngo sman |
| *Pedicularis decorisima* Diels | *Pedicularis decorissima*Diels | Scrophulariaceae | lug ru dmar po | sngo ldum | sngo sman |
| *Pedicularis elwesii* Hk. f. | *Pedicularis elwesii*Hook. f. | Scrophulariaceae | lha glang | sngo ldum | sngo sman |
| *Pedicularis integrifolia* Hk.f | *Pedicularis integrifolia*Hook. f. | Scrophulariaceae | ma ning glang sna | sngo ldum | sngo sman |
| *Pedicularis lachnoglossa* Hk.f. | *Pedicularis lachnoglossa*Hook. f. | Scrophulariaceae | 'dre glang | sngo ldum | sngo sman |
| *Pedicularis lineata* Franch. ex Maxim. | *Pedicularis lineata*Franch. ex Maxim. | Scrophulariaceae | mo glang | sngo ldum | sngo sman |
| *Pedicularis longiflora* Rudolph var. *tubiformis* (Klotz. ) Tsoong | *Pedicularis longiflora* Rudolph | Scrophulariaceae | lug ru ser po | sngo ldum | sngo sman |
| *Pedicularis megalochila* Li. | *Pedicularis megalochila*H.L. Li | Scrophulariaceae | pho glang | sngo ldum | sngo sman |
| *Pedicularis trichoglossa* Hk. f. | *Pedicularis trichoglossa* Hook. f. | Scrophulariaceae | sngo bong dmar | sngo ldum | sngo sman |
| *Pegaeophyton scapiflorum* (J. D. Hooker et Thomson) C. Marquand et Airy Shaw | *Pegaeophyton scapiflorum*(Hook.f. & Thomson) C.Marquand & Airy Shaw | Brassicaceae | sro lo dkar po | sngo ldum | sngo sman |
| *Pennisetum flaccidum* Griseb. | *Pennisetum flaccidum*Griseb. | Poaceae | dUr wa | sngo ldum | sngo sman |
| *Petasites tricholobus* Franch. | *Petasites tricholobus*Franch. | Asteraceae | lug sho | sngo ldum | ldum bu thang sman |
| *Phaseolus lunatus* Billb. ex Beurl./ *Phaseolus lunatus* L. | *Phaseolus lunatus*L. | Fabaceae | mkhal zho dkar po | shing sman | shing sman |
| *Phaseolus vulgaris* L. | *Phaseolus vulgaris* L. | Fabaceae | mon sran gre'u | zhing skyes 'bru'i khal | zhing skyes 'bru'i khal |
| *Phlomis betonicoides* f. alba C.Y.Wu | *Phlomis betonicoides* f. alba C.Y.Wu | Lamiaceae | pu shud mig sman | sngo ldum | sngo sman |
| *Phlomis younghusbandii* Mukerj. | *Phlomis younghusbandii*Mukerjee | Lamiaceae | lug mur | sngo ldum | sngo sman |
| *Phoenix dactylifera* L. | *Phoenix dactylifera* L. | Arecaceae | 'bra go | shing sman | shing sman |
| *Phyllanthus emblica* L. | *Phyllanthus emblica* L. | Euphorbiaceae | skyu ru ra | shing sman | shing sman |
| *Phyllostachys glauca* McClure | *Phyllostachys glauca*McClure | Poaceae | smyug sngon tshi ba | shing sman | sngo sman |
| *Physochlaina praealta* (Decne.) Miers | *Physochlaina praealta*(Decne.) Miers | Solanaceae | ser po d+ha du ra | sngo ldum | ldum bu thang sman |
| *Phytolacca acinosa* Roxb. | *Phytolacca acinosa*Roxb. | Phytolaccaceae | dpa' bo | sngo ldum | sngo sman |
| *Picea brachytyla*（Franch.）Pritz. | *Picea brachytyla*(Franch.) E.Pritz. | Pinaceae | thang shing | shing sman | shing sman |
| *Picris hieracioides* L. | *Picris hieracioides*Sibth. & Sm. | Asteraceae | rgya khur dkar po | sngo ldum | sngo sman |
| *Pinellia ternata*（Thunb.）Breit. | *Pinellia ternata*(Thunb.) Makino | Araceae | dwa g.yung | sngo ldum | sngo sman |
| *Pinus tabulaeformis* Carr. | *Pinus tabuliformis*Carrière | Pinaceae | sgron shing | shing sman | shing sman |
| *Piper cubeba* L. | *Piper cubeba* L. | Piperaceae | rin chen smyag | shing sman | shing sman |
| *Piper longum* L. | *Piper longum*L. | Piperaceae | pi pi ling | shing sman | shing sman |
| *Piper nigrum* L. | *Piper nigrum*L. | Piperaceae | na le sham | shing sman | shing sman |
| *Piper nigrum* L. | *Piper nigrum*L. | Piperaceae | pho ba ris | shing sman | shing sman |
| *Piptanthus nepalensis* (Hook.) D. Don | *Piptanthus nepalensis*(Hook.) D.Don | Fabaceae | dang ma ser po | sngo ldum | sngo sman |
| *Pisum sativum* Linn. | *Pisum sativum*L. | Fabaceae | srad ma ril mo | zhing skyes 'bru'i khal | zhing skyes 'bru'i khal |
| *Plantago depressa* Willd. | *Plantago depressa*Willd. | Plantaginaceae | tha ram | sngo ldum | sngo sman |
| *Plantago major* L. | *Plantago major* L. | Plantaginaceae | na skyes tha ram | sngo ldum | sngo sman |
| *Platycladus orientalis*（L.）Franco | *Platycladus orientalis*(L.) Franco | Cupressaceae | rgya shug leb mo | shing sman | shing sman |
| *Pleurospermum amabile* Craib ex W.W.Smith. | *Pleurospermum amabile*W. G. Craib & W.W. Sm. | Apiaceae | rtsad mchog | sngo ldum | sngo sman |
| *Pleurospermum hookeri* C.B.Clarke var. *thomsonii* C.B.Clarke. | *Pleurospermum hookeri*C.B. Clarke | Apiaceae | rtsad mo rigs | sngo ldum | sngo sman |
| *Pleurospermum wrightianum* de Boiss. | *Pleurospermum wrightianum*H. Boissieu | Apiaceae | rtsad | sngo ldum | sngo sman |
| *Pluteus cervinus*(Schaeff.) P. Kumm. | *Pluteus cervinus*(Schaeff.) P. Kumm. | Pluteaceae | bye'u sha mo | sngo ldum | sngo sman |
| *Polygonatum cirrhifolium* (Wall.) Royle. | *Polygonatum cirrhifolium*(Wall.) Royle | Liliaceae | ra mnye | sngo ldum | ldum bu thang sman |
| *Polygonatum prattii* Baker | *Polygonatum pratti*i Baker | Liliaceae | lug mnye | sngo ldum | ldum bu thang sman |
| *Polygonum calostachyum* Diels. | *Polygonum calostachyum* Diels. | Polygonaceae | ga dur dman pa | sngo ldum | sngo sman |
| *Polygonum hookeri* Meisn. | *Polygonum hookeri* Meisn. | Polygonaceae | chu rtsi g.yung ba | sngo ldum | ldum bu thang sman |
| *Polygonum macrophyllum* D. Don var. *stenophyllum* (Meisn.) A. J. Li | *Polygonum macrophyllum*D. Don | Polygonaceae | mon bu | sngo ldum | sngo sman |
| *Polygonum macrophyllum* D.Don. | *Polygonum macrophyllum D. Don* | Polygonaceae | gla sgang | sngo ldum | sngo sman |
| *Polygonum sibiricum* Laxm. | *Polygonum sibiricum*Laxm. | Polygonaceae | tsi tsi sa 'dzin | sngo ldum | sngo ldum |
| *Polygonum sinomontanum* Sam. | *Polygonum sinomontanum*Sam. | Polygonaceae | rta mon pa | sngo ldum | sngo sman |
| *Polygonum tortuosum* D. Don | *Polygonum tortuosum*D. Don | Polygonaceae | snya lo | sngo ldum | ldum bu thang sman |
| *Polygonum viviparum* L. | *Polygonum viviparum* L. | Polygonaceae | ram bu | sngo ldum | sngo sman |
| *Polystichum squarrosum* (Don) Fee. | *Polystichum squarrosum*(D. Don) Fée | Dryopteridaceae | g.yu 'brug 'khyil ba | sngo ldum | sngo sman |
| *Pomatosace filicula* Maxim. | Pomatosace filicula Maxim. | Primulaceae | re skon sga tig | sngo ldum | sngo sman |
| *Populus tomentosa* Carr. | *Populus  tomentosa*Carrière | Salicaceae | dbyar pa | shing sman | shing sman |
| *Populus alba* L. | *Populus alba*L. | Salicaceae | ma gal | shing sman | shing sman |
| *Populus davidiana* Dode. | *Populus davidiana* Dode. | Salicaceae | sheng de wa | shing sman | shing sman |
| *Populus qamdoensis* C. Wang et Tung | *Populus qamdoensis*C. Wang & S.L. Tung | Salicaceae | lcang ma | shing sman | shing sman |
| *Potentilla anserina* L. | *Potentilla anserina*L. | Rubiaceae | gro ma | sngo ldum | sngo sman |
| *Potentilla fruticosa* L. | *Potentilla fruticosa* L. | Rubiaceae | spen nag | shing sman | shing sman |
| *Potentilla fruticosa* L. var. *pumila* Hook. f. | *Potentilla fruticosa* L. var. *pumila* Hook. f. | Rubiaceae | spen chung | shing sman | shing sman |
| *Potentilla glabra* Lodd. | *Potentilla glabra* G.Lodd. | Rubiaceae | spen dkar | shing sman | shing sman |
| *Primula bracteata* Franch. | *Primula bracteata*Franch. | Primulaceae | bdud rtsi sga tig | sngo ldum | sngo sman |
| *Primula calliantha* Franch. subsp. *bryophila* (I.B.Balfour G Forrest) W.W. Smith & Forrest. | *Primula calliantha* subsp. *bryophila*(Balf. f. & Farrer) W.W. Sm. & Forrest | Primulaceae | shang dril smug chung | sngo ldum | sngo sman |
| *Primula chionantha* I. B. Balfour & Forrest | *Primula chionantha Balf. f. & Forrest* | Primulaceae | shang dril smug po | sngo ldum | sngo sman |
| *Primula denticulata* Smith | *Primula denticulata* Sm. | Primulaceae | sha pho rgyu ma | sngo ldum | sngo sman |
| *Primula fasciculata* Balf. f. et Ward | *Primula fasciculata Balf. f. & Kingdon-Ward* | Primulaceae | g.yar mo thang pa | sngo ldum | sngo sman |
| *Primula florindae* Ward | *Primula florindae Kingdon-Ward* | Primulaceae | shang dril ser po | sngo ldum | sngo sman |
| *Primula littledalei* Balf. f. et Watt | *Primula littledalei*Balf. f. & Watt | Primulaceae | brag lcam dri zhim | sngo ldum | sngo sman |
| *Primula secundiflora* Franch | *Primula secundiflora Franch.* | Primulaceae | shang dril dmar po | sngo ldum | sngo sman |
| *Primula sikkimensis* Hook.F | *Primula sikkimensis Hook.* | Primulaceae | shang dril | sngo ldum | sngo sman |
| *Prunus davidiana*（Carr）Franch. | *Prunus davidiana*(CarriŠre) Franch. | Rubiaceae | ri kham | shing sman | shing sman |
| *Prunus mira* Koehne | *Prunus mira*Koehne | Rubiaceae | bod kham | shing sman | shing sman |
| *Prunus persica*（L.）Batsch | *Prunus persica* (L.) Batsch | Rubiaceae | kham bu | shing sman | shing sman |
| *Przewalskia tangutica* Maxim. | *Przewalskia tangutica*Maxim. | Solanaceae | thang phrom dkar po | sngo ldum | ldum bu thang sman |
| *Pterocarpus santalinus* L. f. | *Pterocarpus santalinus*L.f. | Fabaceae | tsan dan dmar po | shing sman | shing sman |
| *Pterocephalus bretschneideri* (Bat.) Pritz. | *Pterocephalus bretschneideri*(Batalin) E. Pritz. | Dipsacaceae | lug rtsi do bo | sngo ldum | sngo sman |
| *Pterocephalus hookeri*（C. B. Clarke）Hock. | *Pterocephalus hookeri*(C.B.Clarke) E.Pritz. | Dipsacaceae | spang rtsi do bo | sngo ldum | sngo sman |
| *Pulicaria insignis* Drumm. ex Dunn | *Pulicaria insignis*Drumm*.* ex Dunn | Asteraceae | ming can | sngo ldum | ldum bu thang sman |
| *Punica granatum* L. | *Punica granatum*L. | Punicaceae | se 'bru | shing sman | shing sman |
| *Quercus aquifolioides* Rehd. et Wils. | *Quercus aquifolioides*Rehder & E.H.Wilson | Fagaceae | mon cha ra | shing sman | shing sman |
| *Rabdosia pseudoirrorata* C. Y. Wu | *Rabdosia pseudoirrorata* C. Y. Wu | Lamiaceae | phur mong nag po | sngo ldum | ldum bu thang sman |
| *Ramaria botrytoides* (Peck) Corner. | *Ramaria botrytoides* (Peck) Corner. | Clavariaceae | bya lag bye'u sug | sngo ldum | sngo sman |
| *Ranunculus involucratus* Maxim. | *Ranunculus involucratus* Maxim. | Ranunculaceae | lce tsha gab skyes | sngo ldum | sngo sman |
| *Ranunculus longicaulis* C. A. Mey. var. *nephelogenes* (Edgew.) L. Liu | *Ranunculus longicaulis*Ledeb. ex A.Spreng. | Ranunculaceae | sga tsha | sngo ldum | sngo sman |
| *Ranunculus minor* (L. Liou) W.T. Wang | *Ranunculus minor*(L. Liou) W.T. Wang | Ranunculaceae | lce tsha gab chung | sngo ldum | sngo sman |
| *Ranunculus pegaeus* Hand.-Mazz. | *Ranunculus pegaeus*Hand.-Mazz. | Ranunculaceae | lce tsha sa 'dzin | sngo ldum | sngo sman |
| *Ranunculus tanguticus* (Maxim.) Ovcz. | *Ranunculus tanguticus* (Maxim.) Ovcz. | Ranunculaceae | 'bri mo lce tsha | sngo ldum | sngo sman |
| *Raphanus sativus* L. | *Raphanus sativus* L. | Brassicaceae | la phug | zhing skyes 'bru'i khal | zhing skyes 'bru'i khal |
| *Rhamnella gilgitica* Mansf. et Melch. | *Rhamnella gilgitica*Mansf. & Melch. | Rhamnaceae | seng ldeng | shing sman | shing sman |
| *Rheum nobile* Hook.f.et Thoms. | *Rheum nobile*Hook. f. & Thomson | Polygonaceae | chu ma rtsi | sngo ldum | ldum bu thang sman |
| *Rheum officinale* Baill. | *Rheum officinale*Baill. | Polygonaceae | lcum rtsa | sngo ldum | ldum bu thang sman |
| *Rheum pumilum* Maxim. | *Rheum pumilum*Maxim. | Polygonaceae | chu rtsi rgod pa | sngo ldum | ldum bu thang sman |
| *Rheum spiciforme* Royle. | *Rheum spiciforme*Royle | Polygonaceae | chu rtsa | sngo ldum | ldum bu thang sman |
| *Rheum webbianum* Royle | *Rheum webbianum*Royle | Polygonaceae | la lcum | sngo ldum | ldum bu thang sman |
| *Rhodiola chrysanthemifolia*（Levl.）S. H. Fu | *Rhodiola chrysanthemifolia*(H. Lév.) S.H. Fu | Crassulaceae | 'dre tshan skya bo | sngo ldum | sngo sman |
| *Rhodiola crenulata* (Hk. f. et Thoms.) H. Ohba | *Rhodiola crenulata*(Hook.f. & Thomson) H.Ohba | Crassulaceae | sro lo dmar po | sngo ldum | sngo sman |
| *Rhodiola dumulosa* (Franch.) S. H. Fu | *Rhodiola dumulosa*(Franch.) S.H. Fu | Crassulaceae | gangs tshan pa | sngo ldum | sngo sman |
| *Rhodiola fastigiata*（Hook. f. et. Thoms.）S. H. Fu | *Rhodiola fastigiata*(Hook. f. & Thomson) S.H. Fu | Crassulaceae | lha tshan dmar po | sngo ldum | sngo sman |
| *Rhodiola himalensis*（D. Don）S. H. Fu. | *Rhodiola himalensis*(D. Don) S.H. Fu | Crassulaceae | ma ning tshan dmar | sngo ldum | sngo sman |
| *Rhodiola kirilowii* (Regel) Maxim. | *Rhodiola kirilowii*(Regel) Maxim. | Crassulaceae | spang tshan pa | sngo ldum | sngo sman |
| *Rhodiola prainii*（Hamet）H. Ohba | *Rhodiola prainii*(Raym.-Hamet) H. Ohba | Crassulaceae | brag tshan pa | sngo ldum | sngo sman |
| *Rhodiola quadrifida* (Pall.) Fisch. et. Mey. | *Rhodiola quadrifida* (Pall.) Fisch. et. Mey. | Crassulaceae | chu tshan pa | sngo ldum | sngo sman |
| *Rhodiola sacra*（Prain ex Hamet）S. H. Fu | *Rhodiola sacra*(Prain ex Raym.-Hamet) S.H. Fu | Crassulaceae | g.ya' tshan pa | sngo ldum | sngo sman |
| *Rhodiola serrata* H. Ohba | *Rhodiola serrata*H. Ohba | Crassulaceae | tshan dmar pho ldum | sngo ldum | sngo sman |
| *Rhodiola smithii* (Hamet) S. H. Fu | *Rhodiola smithii* (Raym.-Hamet) S.H. Fu | Crassulaceae | brag lcam pa | sngo ldum | sngo sman |
| *Rhodiola smithii* (Hamet) S. H. Fu | *Rhodiola smithii* (Raym.-Hamet) S.H. Fu | Crassulaceae | tshan dmar mo ldum | sngo ldum | sngo sman |
| *Rhododendron anthopogonoides* Maxim. | *Rhododendron anthopogonoides*Maxim. | Ericaceae | da li dri zhim | shing sman | shing sman |
| *Rhododendron nivale* Hook. f. | *Rhododendron nivale*Hook. f. | Ericaceae | da li nag po | shing sman | shing sman |
| *Rhododendron phaeochrysum* Balf. f. et W. W. Smith. | *Rhododendron phaeochrysum*Balf. f. & W.W. Sm. | Ericaceae | stag ma | shing sman | shing sman |
| *Rhododendron primuliflorum* Bur. et Franch. | *Rhododendron primuliflorum*Bureau & Franch. | Ericaceae | da li | shing sman | shing sman |
| *Rhododendron wightii* Hook. f. | *Rhododendron wightii*Hook. f. | Ericaceae | stag ma ser po | shing sman | shing sman |
| *Rhus chinensis* Mill. | *Rhus chinensis*Mill. | Anacardiaceae | da trig | shing sman | shing sman |
| *Ribes alpestre* Wall. ex Decne. | *Ribes alpestre*Wall. ex Decne. | Saxifragaceae | se'u nag | shing sman | shing sman |
| *Ribes himalense* Royle ex Decne. | *Ribes himalense*Royle ex Decne. | Saxifragaceae | se'u shing | shing sman | shing sman |
| *Ricinus communis* L. | *Ricinus communis* L. | Euphorbiaceae | dan+da khra bo | shing sman | shing sman |
| *Rorippa indica* (L.) Hiern | *Rorippa indica* (L.) Hiern | Brassicaceae | ske tshe | sngo ldum | sngo sman |
| *Rosa omeiensis* Rolfe | *Rosa omeiensis* Rolfe | Rubiaceae | se ba | shing sman | shing sman |
| *Rosa rubus* Lévl. et Vant | *Rosa rubus* H.Lv. & Vaniot | Rubiaceae | se rgod mo | shing sman | shing sman |
| *Rosa sertata* Rolfe | *Rosa sertata*Rolfe | Rubiaceae | se rgod | shing sman | shing sman |
| *Rubia chinensis* Regel et Maack | *Rubia chinensis*Regel & Maack | Rubiaceae | ma thang 'ching bu nag po | sngo ldum | sngo sman |
| *Rubia manjith* Roxb. ex Flem | *Rubia manjith*Roxb. ex Fleming | Rubiaceae | btsod | shing sman | shing sman |
| *Rubus ellipticus* Smith | *Rubus ellipticus* Sm. | Rubiaceae | kaN+Da ka ri | shing sman | shing sman |
| *Rubus irritans* Focke | *Rubus irritans*Focke | Rubiaceae | ga bra chung ba | shing sman | shing sman |
| *Rubus subornatus* Focke | *Rubus subornatus*Focke | Rubiaceae | ga bra | shing sman | shing sman |
| *Rumex acetosella* L. | *Rumex acetosella*L. | Polygonaceae | mkha' 'gro la phug | sngo ldum | ldum bu thang sman |
| *Rumex crispus* L. | *Rumex crispus*L. | Polygonaceae | rgya sho | sngo ldum | ldum bu thang sman |
| *Rumex nepalensis* Spreng. | *Rumex nepalensis*Spreng. | Polygonaceae | sho mang | sngo ldum | ldum bu thang sman |
| *Salix alba* L. | *Salix alba*L. | Salicaceae | klung lcang skyed ma | shing sman | shing sman |
| *Salix babylonica* L. | *Salix babylonica*L. | Salicaceae | rgya lcang phra mo | shing sman | shing sman |
| *Salix oritrepha* Schneid. | *Salix oritrepha*C.K. Schneid. | Salicaceae | glang ma dkar po | shing sman | shing sman |
| *Salix sclerophylla* Anderss | *Salix sclerophylla*Andersson | Salicaceae | glang ma | shing sman | shing sman |
| *Salvia prattii* Hemsl. | *Salvia prattii* Hemsl. | Lamiaceae | 'jib rtsi chen po | sngo ldum | sngo sman |
| *Salvia przewalskii* Maxim. | *Salvia przewalskii*Maxim. | Lamiaceae | 'jib rtsi smug po | sngo ldum | sngo sman |
| *Salvia roborowskii* Maxim. | *Salvia roborowskii*Maxim. | Lamiaceae | zhim thig ser po | sngo ldum | sngo sman |
| *Santalum album* L. | *Santalum album*L. | Santalaceae | tsan dan dkar po | shing sman | shing sman |
| *Sapindus mukorossi* Gaertn. | *Sapindus mukorossi*Gaertn. | Sapindaceae | lung tang | shing sman | shing sman |
| *Sarcodon imbricatum* (L.ex Fr.) Karst. | *Sarcodon imbricatum* (L.ex Fr.) P. Karst. | Bankeraceae | shing gi sha mong | sngo ldum | sngo sman |
| *Saussurea graminea* Dunn | *Saussurea graminea*Dunn | Asteraceae | rtsa mkhris ba mo kha | sngo ldum | sngo sman |
| *Saussurea hieracioides* Hook. f. | *Saussurea hieracioides*Hook.f. | Asteraceae | bai Dur+ya 'dra | sngo ldum | sngo sman |
| *Saussurea katochaetoides* Hand.-Mazz. | *Saussurea katochaetoides* Hand.-Mazz. | Asteraceae | khyung sder dkar po | sngo ldum | sngo sman |
| *Saussurea kingii* C.E.C.Fisch. | *Saussurea kingii*J.R.Drumm. ex C.E.C.Fisch. | Asteraceae | bya rog nyungs ma | sngo ldum | sngo sman |
| *Saussurea medusa* Maxim. | *Saussurea medusa*Maxim. | Asteraceae | bya rgod sug ba | sngo ldum | sngo sman |
| *Saussurea obvallata* (DC.) Edgew. | *Saussurea obvallata*(DC.) Edgew. | Asteraceae | gza' dug nag po | sngo ldum | sngo sman |
| *Saussurea pachyneura* Franch. | *Saussurea pachyneura*Franch. | Asteraceae | kon pa gab chung | sngo ldum | sngo sman |
| *Saussurea paxiana* Diels | *Saussurea paxiana*Diels ex H.Limpr. | Asteraceae | rdza yi sur snel | sngo ldum | sngo sman |
| *Saussurea przewalskii* Maxim. | *Saussurea przewalskii*Maxim. | Asteraceae | kon pa gab skyes | sngo ldum | sngo sman |
| *Saussurea stella* Maxim. | *Saussurea stella*Maxim. | Asteraceae | khyung sder smug po | sngo ldum | sngo sman |
| *Saxifraga egregia* Engl. | *Saxifraga egregia* Engl. | Saxifragaceae | 'od ldan | sngo ldum | sngo sman |
| *Saxifraga melanocentra* Franch. | *Saxifraga melanocentra*Franch. | Saxifragaceae | 'od ldan dkar po | sngo ldum | sngo sman |
| *Saxifraga nigroglandulifera* Balakr. | *Saxifraga nigroglandulifera*N.P. Balakr. | Saxifragaceae | gur tig | sngo ldum | ldum bu thang sman |
| *Saxifraga punctulata* Engl. | *Saxifraga punctulata* Engl. | Saxifragaceae | sum tig dkar po | sngo ldum | ldum bu thang sman |
| *Saxifraga sinomontana* J. T. Pan & Gornall. | *Saxifraga sinomontana*J.T. Pan & Gornall | Saxifragaceae | sum tig chung ba | sngo ldum | ldum bu thang sman |
| *Saxifraga tangutica* Engl. | *Saxifraga tangutica*Engl. | Saxifragaceae | gser tig dkar po | sngo ldum | ldum bu thang sman |
| *Saxifraga umbellulata* Hook. f. et Thoms. var. *pectinata* (Marquand et Airy-Shaw) J. T. Pan. | *Saxifraga umbellulata*Hook. f. & Thomson | Saxifragaceae | sum cu tig ta | sngo ldum | ldum bu thang sman |
| *Scrophularia buergeriana* Miq. | *Scrophularia buergeriana*Miq. | Berberidaceae | g.yer shing mchog | shing sman | shing sman |
| *Scrophularia dentata* Royle ex Benth. | *Scrophularia dentata*Royle ex Benth. | Berberidaceae | g.yer shing pa | sngo ldum | ldum bu thang sman |
| *Scrophularia spicata* Franch. | *Scrophularia spicata*Franch. | Berberidaceae | g.yer shing g.yung ba | sngo ldum | ldum bu thang sman |
| *Sedum bulbiferum* Makino | *Sedum bulbiferum Makino* | Crassulaceae | gnyan thub pa | sngo ldum | sngo sman |
| *Selaginella pulvinata* (Hook. et Grev.) Maxim. | *Selaginella pulvinata (Hook. & Grev.)* Maxim. | Selaginellaceae | sbal pa lag pa | sngo ldum | sngo sman |
| *Semecarpus anacardius* L. f. | *Semecarpus anacardius* L. f. | Anacardiaceae | go bye | shing sman | shing sman |
| *Semen Sesami* Nigrum | *Semen Sesami* Nigrum | Pedaliaceae | til nag | zhing skyes 'bru'i khal | zhing skyes 'bru'i khal |
| *Senecio thianschanicus* Regel & Schmalhausen. | *Senecio thianschanicus* Regel & Schmalh. | Asteraceae | sga chung gser mgo | sngo ldum | ldum bu thang sman |
| *Senna bicapsularis* (L.) Roxb. | *Senna bicapsularis (L.) Roxb.* | Fabaceae | shing thal ka rdo rje | sngo ldum | sngo sman |
| *Senna tora* Linn. | *Senna tora*(L.) Roxb. | Fabaceae | thal ka rdo rje | sngo ldum | sngo sman |
| *Sesamum indicum* L. | *Sesamum indicum*L. | Pedaliaceae | til | zhing skyes 'bru'i khal | zhing skyes 'bru'i khal |
| *Setaria italica* (L.)Beauv. | *Setaria italica*(L.) P.Beauv. | Poaceae | khre | zhing skyes 'bru'i khal | zhing skyes 'bru'i khal |
| *Sibiraea angustata* (Rehd.) Hand.-Mazz. | *Sibiraea angustata*(Rehder) Hand.-Mazz. | Rubiaceae | nya brid | shing sman | shing sman |
| *Silene kantzeensis* C. L. Tang | *Silene kantzeensis* C. L. Tang | Caryophyllaceae | ger skyes dmar po | sngo ldum | sngo sman |
| *Silene lhassana* (Williams) Majumdar | *Silene lhassana*(F.N. Williams) C.L. Tang | Caryophyllaceae | lug sug chung ba | sngo ldum | sngo sman |
| *Silene waltoni* Williams | *Silene waltoni* Williams | Caryophyllaceae | ra sug | sngo ldum | sngo sman |
| *Silene yetii B*ocquet | *Silene yetii*Bocquet | Caryophyllaceae | sug pa | sngo ldum | sngo sman |
| *Sinapis alba* L. | *Sinapis alba* L. | Brassicaceae | yungs dkar | zhing skyes 'bru'i khal | zhing skyes 'bru'i khal |
| *Sinocarum coloratum* （Diels）H. Wolff | *Sinocarum coloratum* (Diels) H. Wolff ex Shan & F.T. Pu | Apiaceae | tang kun dkar po | sngo ldum | sngo sman |
| *Sinolimprichtia alpina* Wolff | *Sinolimprichtia alpina*H. Wolff | Apiaceae | tang kun | sngo ldum | sngo sman |
| *Sinopodophyllum hexandrum*（Royle）Ying | *Sinopodophyllum hexandrum*(Royle) T.S.Ying | Berberidaceae | 'ol mo se | sngo ldum | sngo ldum |
| *Sisymbrium heteromallum* C. A. Mey. | *Sisymbrium heteromallum*C.A. Mey. | Brassicaceae | sgang thog pa | sngo ldum | sngo sman |
| *Skimmia multinervia* Huang | *Skimmia multinervia*C.C. Huang | Rutaceae | nags zhun | shing sman | shing sman |
| *Solms-Laubachia eurycarpa* (Maxim) Botsch. | *Solms-laubachia eurycarpa*(Maxim.) Botsch. | Brassicaceae | sro lo smug po | sngo ldum | sngo sman |
| *Solms-Laubachia lanata* Botsch. | *Solms-laubachia lanata*Botsch. | Brassicaceae | srol dkar spra 'dra | sngo ldum | sngo sman |
| *Sonchus brachyotus* DC. | *Sonchus brachyotus*DC. | Asteraceae | rgya khur nag po | sngo ldum | sngo sman |
| *Sophora davidii* (Franch.) Skeels. | *Sophora davidii*(Franch.) Pavol. | Fabaceae | skyi ba'i 'bras bu | shing sman | shing sman |
| *Sophora moorcroftiana* (Benth.) Baker | *Sophora moorcroftiana (Benth.)* Baker | Fabaceae | skyi tsher | shing sman | shing sman |
| *Sorbus rehderiana* Koehne | *Sorbus rehderiana*Koehne | Rubiaceae | rma mo | shing sman | shing sman |
| *Sorbus rufopilosa* Schneid. | *Sorbus rufopilosa* C.K.Schneid. | Rubiaceae | rma mo chung ba | shing sman | shing sman |
| *Soroseris hookeriana* (C.B.Clarke) Stebb subsp. erysimoides Stebb. | *Soroseris hookeriana (C.B.Clarke) Stebbins* | Asteraceae | srol gong pa | sngo ldum | sngo sman |
| *Spenceria ramalana* Trimen | *Spenceria ramalana*Trimen | Rubiaceae | 'bal mo spang dga' | sngo ldum | sngo sman |
| *Spiraea canescens* D. Don | *Spiraea canescens*D.Don | Rubiaceae | smag chung | shing sman | shing sman |
| *Spiraea schneideriana* Rehd. | *Spiraea schneideriana*Rehder | Rubiaceae | smag shad | shing sman | shing sman |
| *Spirogyra intorta* Jao. | *Spirogyra intorta* Jao. | Zyganemataceae | nya lcibs | sngo ldum | sngo sman |
| *Stebbinsia umbrella* (Franch.) Lipsch | *Stebbinsia umbrella* (Franch.) Lipsch | Asteraceae | rdza skyes srol gong | sngo ldum | sngo sman |
| *Stellera chamaejasme* Linn. | *Stellera chamaejasme*L. | Thymelaeaceae | re lcag pa | sngo ldum | ldum bu thang sman |
| *Stephania cephalantha* Hayata | *Stephania cephalantha*Hayata | Ericaceae | a sbi rdo lo | sngo ldum | shing sman |
| *Strychnos nux-vomica* Linn. | *Strychnos nux-vomica*L. | Loganiaceae | ko byi la | shing sman | shing sman |
| *Stylophorum lasiocarpum*（Oliv.）Fedde | *Stylophorum lasiocarpum*(Oliv.) Fedde | Papaveraceae | me tog gser chen | sngo ldum | sngo sman |
| *Swertia chirayita* Buch.-Ham | *Swertia chirayita* Buch.-Ham | Gentianaceae | tig ta | sngo ldum | ldum bu thang sman |
| *Swertia ciliata* （D.Don ex G. Don）B. L. Burtt | *Swertia ciliata*(D. Don ex G. Don) B.L. Burtt | Gentianaceae | bal tig | sngo ldum | ldum bu thang sman |
| *Swertia franchetiana* H. Smith. | *Swertia franchetiana*Harry Sm. | Gentianaceae | dngul tig | sngo ldum | ldum bu thang sman |
| *Swertia hispidicalyx* Burk | *Swertia hispidicalyx*Burkill | Gentianaceae | zangs tig dman pa | sngo ldum | ldum bu thang sman |
| *Swertia mussotii* Franch. | *Swertia mussotii*Franch. | Gentianaceae | zangs tig | sngo ldum | ldum bu thang sman |
| *Swertia racemosa* （Griseb.）Wall ex C. B. Clarke | *Swertia racemosa*(Wall. ex Griseb.) C.B. Clarke | Gentianaceae | rgya tig rnam gcig | sngo ldum | ldum bu thang sman |
| *Swertia verticillifolia* T. N. Ho et S. W. Liu. | *Swertia verticillifolia*T.N. Ho & S.W. Liu | Gentianaceae | dpa' bo ser po | sngo ldum | sngo sman |
| *Swertia wardii* C. Marquand | *Swertia wardii C.* Marquand | Gentianaceae | ser po rgu drus | sngo ldum | sngo sman |
| *Symplocos paniculata*（Thunb.）Miq. | *Symplocos paniculata*（Thunb.）Miq. | Symplocaceae | zhu mkhan | shing sman | shing sman |
| *Syncalathium kawaguchii* (Kitam.) Ling | *Syncalathium kawaguchii*(Kitam.) Ling | Asteraceae | srol gong smug po | sngo ldum | sngo sman |
| *Synotis solidaginea* (Hand.-Mazz.) C. Jeffrey et Y. L. Chen | *Synotis solidaginea* (Hand.-Mazz.) C.Jeffrey & Y.L.Chen | Asteraceae | yu gu shing | sngo ldum | ldum bu thang sman |
| *Syzygium cumini* ( L.) Skeels | *Syzygium cumini*(L.) Skeels | Myrtaceae | sra 'bras | shing sman | shing sman |
| *Tagetes erecta* L. | *Tagetes erecta*L. | Asteraceae | le brgan | sngo ldum | sngo sman |
| *Takakia lepidozioides* Hatt. | *Takakia lepidozioides*S. Hatt. & Inoue | Takakiaceae | grog shing | sngo ldum | shing sman |
| *Taraxacum sikkimense* Hand.-Mazz. | *Taraxacum sikkimense*Hand.-Mazz. | Asteraceae | khur dkar | sngo ldum | sngo sman |
| *Taraxacum tibetanum* Hand.-Mazz. | *Taraxacum tibetanum*Hand.-Mazz. | Asteraceae | khur nag | sngo ldum | sngo sman |
| *Taxus wallichiana* Zucc*.* | *Taxus wallichiana*Zucc. | Taxaceae | gsom seng ldeng | shing sman | shing sman |
| *Tephroseris rufa* (Hand.-Mazz.) B. Nord. | *Tephroseris rufa*(Hand.-Mazz.) B.Nord. | Asteraceae | a byag g.yung ba | sngo ldum | sngo sman |
| *Terminalia bellirica* (Gaertn.) Roxb. | *Terminalia bellirica*(Gaertn.) Roxb. | Combretaceae | ba ru ra | shing sman | shing sman |
| *Terminalia chebula* Retz | *Terminalia chebula*Retz | Combretaceae | mchu snyung | shing sman | shing sman |
| *Terminalia chebula* Retz | *Terminalia chebula*Retz | Combretaceae | a ru ra | shing sman | shing sman |
| *Terminalia chebula* Retz | *Terminalia chebula*Retz | Combretaceae | rnam par rgyal ba | shing sman | shing sman |
| *Terminalia chebula* Retz | *Terminalia chebula*Retz | Combretaceae | bdud rtsi sha chen | shing sman | shing sman |
| *Terminalia chebula* Retz | *Terminalia chebula*Retz | Combretaceae | 'phel byed dam bum 'dra | shing sman | shing sman |
| *Terminalia chebula* Retz | *Terminalia chebula*Retz | Combretaceae | skem po | shing sman | shing sman |
| *Terminalia chebula* Retz | *Terminalia chebula*Retz | Combretaceae | 'jigs med | shing sman | shing sman |
| *Thalictrum foliolosum* DC. | *Thalictrum foliolosum*DC. | Ranunculaceae | lcags kyu | sngo ldum | sngo sman |
| *Thalictrum squamiferum* Lecoy. | *Thalictrum squamiferum*Lecoy. | Ranunculaceae | a krong | sngo ldum | ldum bu thang sman |
| *Thalictrum uncatum* Maxim. | *Thalictrum uncatum*Maxim. | Ranunculaceae | ser po khrag rkang | sngo ldum | sngo sman |
| *Thamnolia vermicularis* (Sw.) Ach. ex Schaer | *Thamnolia vermicularis* (Sw.) Ach. ex Schaer | Thamnoliaceae | gangs ga sha ru | sngo ldum | sngo sman |
| *Thermopsis barbata* Benth. | *Thermopsis barbata*Benth. | Fabaceae | gla ba srad ma | sngo ldum | sngo sman |
| *Thermopsis lanceolata* R. Br. | *Thermopsis lanceolata*R.Br. | Fabaceae | dang ma sngon po | sngo ldum | sngo sman |
| *Thlaspi arvense* L. | *Thlaspi arvense*L. | Brassicaceae | bre ga | sngo ldum | sngo sman |
| *Thysanolaena latifolia* (Roxburgh ex Hornemann) Honda | *Thysanolaena latifolia*(Roxb. ex Hornem.) Honda | Poaceae | ku sha | shing sman | sngo sman |
| *Tibetia himalaica* (Baker) Tsui | *Tibetia himalaica* (Baker) H.P.Tsui | Fabaceae | srad smug | sngo ldum | sngo sman |
| *Tinospora cordifolia* (Wulld) Miers | *Tinospora cordifolia* (Wulld) Miers | Ericaceae | sle tres | shing sman | shing sman |
| *Tinospora sinensis* (Lour.) Merr. | *Tinospora sinensis*(Lour.) Merr. | Ericaceae | sle tres | shing sman | shing sman |
| *Torularia humilis* (C. A. Mey.) O. E. Schulz | *Torularia humilis* (C. A. Mey.) O. E. Schulz | Brassicaceae | byi'u la phug | sngo ldum | sngo sman |
| *Toxicodendron vernicifluum* (Stokes) F. A. Barkl | *Toxicodendron vernicifluum* (Stokes) F.A. Barkley | Anacardiaceae | shri khaN+Da | shing sman | shing sman |
| *Trachydium purpurascens* Franch. | *Trachydium purpurascens* Franch. | Apiaceae | 'bam po mo | sngo ldum | ldum bu thang sman |
| *Tribulus terrestris* L. | *Tribulus terrestris*L. | Zygophyllaceae | gze ma | sngo ldum | ldum bu thang sman |
| *Tricholoma matsutake* (lto et lmai) Singer | *Tricholoma matsutake* (lto et lmai) Singer | Tricholomataceae | thang sha dbra pa | sngo ldum | sngo sman |
| *Triglochin maritimum* Linn. | *Triglochin maritimum* Linn. | Potamogetonaceae | na ram | sngo ldum | sngo sman |
| *Trigonella foenum-graecum* Linn. | *Trigonella foenum-graecum*L. | Fabaceae | shu mo za | sngo ldum | sngo sman |
| *Triticum aestivum* L. | *Triticum aestivum*L. | Poaceae | gro | zhing skyes 'bru'i khal | zhing skyes 'bru'i khal |
| *Trollius ranunculoides* Hemsl. | *Trollius ranunculoides* Hemsl. | Ranunculaceae | bong ser | sngo ldum | sngo sman |
| *Ulmus pumila* L. | *Ulmus pumila*L. | Ulmaceae | yo 'bog | shing sman | shing sman |
| *Uncaria scandens*（Smith）Hutchins. | *Uncaria scandens* (Sm.) Hutch. | Rubiaceae | khyung sder | shing sman | shing sman |
| *Urtica hyperborea* Jacq. ex Wedd. | *Urtica hyperborea*Jacq. ex Wedd. | Urticaceae | zwa phyi a ya | sngo ldum | ldum bu thang sman |
| *Urtica tibetica* W. T. Wang | *Urtica tibetica* W. T. Wang | Urticaceae | zwa rgod | sngo ldum | ldum bu thang sman |
| *Urtica triangularis* Hand.-Mazz. | *Urtica triangularis*Hand.-Mazz. | Urticaceae | zwa tshod | sngo ldum | ldum bu thang sman |
| *Urtica triangularis* Hand.-Mazz. subsp. *Pinnatifida* (Hand.-Mazz.) C. J. Chen. | *Urtica triangularis*Hand.-Mazz. | Urticaceae | zwa | sngo ldum | ldum bu thang sman |
| *Usnea diffracta* Vain. | *Usnea diffracta* Vain. | Usneaceae | gser skud | shing sman | shing sman |
| *Ustilago nuda* (Jens) Rostr. | *Ustilago nuda* (Jens) Rostr. | Ustilaginaceae | sa rdzi ka | zhing skyes 'bru'i khal | zhing skyes 'bru'i khal |
| *Valeriana tangutica* Bat | *Valeriana tangutica*Batalin | Valerianaceae | rgya spos | sngo ldum | sngo sman |
| *Veronica eriogyne* H. Winkl. | *Veronica eriogyne*H. Winkl. | Scrophulariaceae | ldum nag dom mkhris | sngo ldum | sngo sman |
| *Vicatia thibetica* de Boiss. | *Vicatia thibetica*H. Boissieu | Apiaceae | lca ba | sngo ldum | sngo sman |
| *Vicia amoena* Fisch. ex DC. | *Vicia amoena Fisch.* | Fabaceae | nya dug pa | sngo ldum | sngo sman |
| *Vicia faba* L. | *Vicia faba*L. | Fabaceae | rgya sran | zhing skyes 'bru'i khal | zhing skyes 'bru'i khal |
| *Vicia unijuga* A.Br. | *Vicia unijuga*A.Br. | Fabaceae | byi'u srad ma | sngo ldum | sngo sman |
| *Viola biflora* L. | *Viola biflora*L. | Violaceae | rta rmig | sngo ldum | sngo sman |
| *Viola kunawarensis* Royle | *Viola kunawarensis* Royle Illustr | Violaceae | ga pa ger skyes | sngo ldum | sngo sman |
| *Vitis vinifera* L. | *Vitis vinifera* L. | Vitaceae | rgun 'brum | shing sman | shing sman |
| *Wikstroemia canescens*（Wall.）Meisn. | *Wikstroemia canescens W*all. ex Meisn. | Thymelaeaceae | shog shing ar nag | shing sman | shing sman |
| *Xanthium strumarium* L. | *Xanthium strumarium*L. | Asteraceae | byis tsher | sngo ldum | sngo sman |
| *Xanthoceras sorbifolium* Bunge | *Xanthoceras sorbifolium*Bunge | Sapindaceae | tsan dan seng ldeng | shing sman | shing sman |
| *Zanthoxylum bungeanum* Maxim. | *Zanthoxylum bungeanum*Maxim. | Rutaceae | g.yer ma | shing sman | shing sman |
| *Zea mays* L. | *Zea mays* L. | Poaceae | a sho pa tra | zhing skyes 'bru'i khal | zhing skyes 'bru'i khal |
| *Zingiber officinale* Rosc | *Zingiber officinale*Roscoe | Zingiberaceae | sga skya | sngo ldum | ldum bu thang sman |
| *Ziziphus jujuba* Mill. var. *inermis* (Bunge) Rehd. | *Ziziphus jujuba*Mill. | Rhamnaceae | 'bra go dmar po | shing sman | shing sman |
| *Ziziphus montana* W.W.Smith | *Ziziphus montana*W.W. Sm. | Rhamnaceae | bo de'i 'bras bu | shing sman | shing sman |
